# Euryale *Small Auxin Up RNA62* promotes cell elongation and seed size by altering the distribution of indole-3-acetic acid under the light

**DOI:** 10.3389/fpls.2022.931063

**Published:** 2022-09-09

**Authors:** Zhi-heng Huang, Ke Bao, Zong-hui Jing, Qian Wang, Hui-fang Duan, Sen Zhang, Wei-wei Tao, Qi-nan Wu

**Affiliations:** ^1^School of Pharmacy, Nanjing University of Chinese Medicine, Nanjing, China; ^2^Jiangsu Collaborative Innovation Center of Chinese Medicinal Resources Industrialization, Nanjing, China

**Keywords:** *Euryale ferox* Salisb., aquatic crop, seed size, *EuSAUR62*, IAA distribution, light

## Abstract

Euryale (*Euryale ferox* Salisb.) is an aquatic crop used as both food and drug in Asia, but its utilization is seriously limited due to low yield. Previously, we hypothesized that *Euryale small auxin up RNA*s (*EuSAUR*s) regulate seed size, but the underlying biological functions and molecular mechanisms remain unclear. Here, we observed that the hybrid Euryale lines (HL) generate larger seeds with higher indole-3-acetic acid (IAA) concentrations than those in the North Gordon Euryale (WT). Histological analysis suggested that a larger ovary in HL is attributed to longer cells around. Overexpression of *EuSAUR62* in rice (*Oryza sativa* L.) resulted in larger glumes and grains and increased the length of glume cells. Immunofluorescence and protein interaction assays revealed that *EuSAUR62* modulates IAA accumulation around the rice ovary by interacting with the rice PIN-FORMED 9, an auxin efflux carrier protein. Euryale basic region/leucine zipper 55 (EubZIP55), which was highly expressed in HL, directly binds to the *EuSAUR62* promoter and activated the expression of *EuSAUR62*. Constant light increased the expression of both *EubZIP55* and *EuSAUR62* with auxin-mediated hook curvature in HL seedlings. Overall, we proposed that *EuSAUR62* is a molecular bridge between light and IAA and plays a crucial role in regulating the size of the Euryale seed.

## Introduction

*Euryale ferox* Salisb. is the only species in the genus Euryale in the botanical family Nymphaeaceae. It is widely distributed in the southern region of China and North Bihar, India, and is considered a staple food because of its high starch content (Jha et al., 2018; Biswas et al., 2020). Biological analysis has revealed that it has antidepression, anti-oxidant, and anti-diabetic pharmacological properties; thus, it is used in traditional Chinese medicines as well (Song et al., 2011; Wu et al., 2017; Huang et al., 2018). However, the low yield of North Gordon Euryale (WT), the most widely distributed type, seriously inhibits the utilization and development of Euryale (Puste et al., 2005). Although all tissues have been developed as medicinal resources, livestock feeds, or non-staple foods, only the seed of Euryale is used as a staple food and as a major source of drugs (Wu et al., 2014). Among the many traits of seed, the size and weight of seed have been proposed as crucial contributors to yield in crop plants (Kesavan et al., 2013; Li and Li, 2015). Recently, we generated a new hybrid Euryale line (HL) with large and heavy seeds, providing a great opportunity to investigate the genetic factors and underlying molecular mechanisms in regulating the seed size of Euryale (Huang et al., 2020).

In China, Euryale is mainly divided into two ecological types: North Gordon Euryale (also known as ‘Ciqian’) and South Gordon Euryale (also known as ‘Suqian’) (Huang et al., 2020). Each Euryale develops ∼18 gigantic floating leaves and –20 obround fruits at the mature stage. The first emersed reproductive tissue occurs 8 or 9 weeks after sowing, until then each plant generates about two reproductive tissues every week upto maturing in the 19th week. Before flowering above water, young reproductive tissue has an underwater developmental period. During this process, the energy and nutrition required for the growth of underwater reproductive tissues should be transported from leaves or stem tubers (Liu et al., 2018; Wu et al., 2022). In this case, both environment and metabolites play crucial roles in controlling Euryale fruit development.

Previous studies have shown that seed development is controlled by the integration of molecular regulatory networks coupled with the spatial and temporal distribution of multiple types of phytohormones, among which auxin is considered a dominant regulator in this process (Li et al., 2019; Cao et al., 2020). The spatial and temporal distribution of active auxin is dynamically mediated by polar auxin transport, biosynthesis, and signaling (Harrison, 2017; Teale and Palme, 2018). In our previous study, we proposed that small auxin up RNA (SAURs) may act as mediators of the auxin signaling pathway and affect the distribution of indole-3-acetic acid (IAA), thereby contributing to the larger seeds of HL (Huang et al., 2020). To date, many genes involved in IAA signal transduction have been documented, such as the *SAUR*, *glycoside hydrolase 3* (*GH3*), and *auxin/indole-3-acetic acid* (*AUX/IAA*) genes, but functional studies on SAURs have lagged behind those of the two primary auxin response genes (Hagen et al., 1984; Abel et al., 1994; Ren and Gray, 2015; Stortenbeker and Bemer, 2019). It has been reported that *AtSAUR62* and *AtSAUR75* play critical roles in pollen tube elongation and subsequent fertilization in *Arabidopsis* (He et al., 2018). AtSAUR63 promotes hypocotyl and stamen filament elongation in *Arabidopsis* (Chae et al., 2012). Auxin is enriched in immature seeds at several stages, especially at the ends of hypophysis and cotyledon primordia during somatic embryo development because of its transportability (Ni et al., 2001). In rice, endogenous IAA concentration also increased in the spikelets after pollination, and this process was positively correlated with IAA synthesis and transport in the ovary (Uchiumi and Okamoto, 2010; Nagasawa et al., 2013). Auxin transport is controlled by several types of influx and efflux carriers, including AUX1/LAX (AUXIN-RESISTANT 1/LIKE-AUX1), NITRATE TRANSPORTER 1.1 (NRT1.1), PIN-FORMED (PIN), PILS (PIN-LIKE), WALLS ARE THIN 1 (WAT1), and ABC transporters (Swarup et al., 2008; Peret et al., 2012; Barbosa et al., 2018). Among them, PIN proteins direct auxin transport by their coordinated polarized localizations to promote auxin accumulation locally and define sites of organogenesis (Zhou and Luo, 2018). In *Arabidopsis thaliana*, eight PIN proteins share two conserved, amino (N)-terminal and carboxyl (C)-terminal domains joined by a less conserved hydrophilic loop (Nodzynski et al., 2016). In maize, ZmPIN1a has been reported to regulate auxin spatiotemporal asymmetric distribution in multiple plant developmental processes (Li et al., 2018). Suppressing apple auxin efflux carrier *MdPIN1* has an impact on leaf shape (Hu et al., 2020). A recent study in *Nymphaea colorata* showed that PIN1, PIN3, and PIN7 are involved in the regulatory network of flower development (Zhang et al., 2020). In *Oryza sativa*, OsPIN9 is monocot-specific PIN protein, and the rice genome contains one further group of two PINs (OsPIN10a and OsPIN10b) (Wang et al., 2009). Monocot-specific and organ-specific proteins exist and they have a distinct role in regulating auxin-induced organ development, such as PIN9 (Balzan et al., 2014). In sorghum, *SbPIN3* and *SbPIN9* are more highly expressed in flowers than in other organs, indicating their roles in regulating the development of reproductive tissues (Shen et al., 2010). Thus, we speculated that both SAURs and PINs may cooperatively regulate IAA-mediated seed size and yield in Euryale.

Normally, in the dark, dicotyledonous plants such as Arabidopsis, rapidly elongate the hypocotyl upward with tightly closed cotyledons, thereby forming an apical hook at the seedling stage, while apical hooks and cotyledons gradually open in response to light (Fankhauser and Chory, 1997; Zadnikova et al., 2016). However, contrary to this established notion, light exaggerates the apical hook curvature in the seedlings of Euryale. Similarly, it has been reported that light causes photoinhibition in *Vallisneria natans* and rice coleoptile, leading to a decrease in plant height and inhibition of coleoptile growth (Riemann et al., 2003; Li H. M. et al., 2020). This response is based on inhibition of cell elongation concomitant with a block of auxin transport (Riemann et al., 2003). We speculated that phototropism stimulates apical curvature formation in Euryale under light conditions according to the theory of polar location of IAA. The inner cells of the hook region become enriched with higher auxin concentration and thereby inhibiting the growth of the inner side during hook formation, while seedlings showed a hookless phenotype when the asymmetrical accumulation of auxin was abolished (Lehman et al., 1996). Thus, the degree of apical curvature in Euryale seedlings under light conditions represents the levels of auxin transport and accumulation. Previous studies provided evidence that light turns off *SAUR17* and *SAUR50* on the inner side of the hook, leading to cell expansion and the opening of the hook, further indicating the role of SAURs in regulating auxin distribution (Lehman et al., 1996; Wang et al., 2020). Some basic leucine zipper (bZIP) proteins that may be able to specifically bind to G-boxes are considered G-box binding factors (GBFs), such as AtbZIP54 and AtbZIP55 (belonging to GBF2 and GBF3, respectively) (Jakoby et al., 2002). *GBF* genes from Arabidopsis are involved in ultraviolet and blue light signal transduction and the regulation of light-responsive promoters (Weisshaar et al., 1991). One cassava *CPRF-2-Like bZIP* gene is up-regulated during white light exposure, indicating its potential function in light response (Pontes et al., 2020). It has been reported that GBF2 and GBF3 are translocated into the nucleus upon light treatment, suggesting that they may act as photoreceptors for phototropism in Euryale seedlings and transactivate *SAURs* (Schindler et al., 1992; Terzaghi et al., 1997).

In this study, we investigated the function of *EuSAUR62* by constructing a gain-of-function strain of rice (*Oryza sativa L.*). The mechanism of *EuSAUR62*-mediated IAA distribution in rice was investigated by detecting the interaction between the EuSAUR62 and OsPIN9 proteins. In addition, differential expression levels of *EuSAUR62* in HL and WT were studied based on the transcriptional regulation of the EubZIP55 on *EuSAUR62*. We also observed light-induced apical curvature to understand the regulation of IAA distribution by EubZIP55 and *EuSAUR62*. Our findings highlighted that *EuSAUR62* plays a crucial role in light-mediated regulation of seed size, which may be a crucial target for improving Euryale yields.

## Materials and methods

### Plant materials

The HL and WT, two varieties of Euryale used in this study, have been described previously (Huang et al., 2020). The plant materials were formally identified by Professor Qinan Wu based on morphology, and a voucher specimen of this material was been deposited at the Nanjing University of Chinese Medicine. Both HL and WT were sown in a completely randomized block design with three replications in the experimental field of Jiangsu Seed and Seed Breeding Base under natural conditions in April 2018. The transplanting period was carried out in early May. Each plot included 160 individual lines, each separated by 2 m from its neighboring lines. During the whole culture process, the water temperature ranges from 21 to 28°C. Before anthesis, young fruits at designed stages (developed from week 9 to week 12) were collected from a single plant and stored at –80°C for histological analysis with three replicates. After fruits ripened in September (the 20th or 21st week), seeds were collected for yield traits, including seed size, seed weight, and 100-seed weight.

A *Japonica* rice variety (*Oryza sativa* L. ssp. *Japonica* cv. Nipponbare) was used for the transgenic experiments. The rice seeds used in this study were sourced from the WT and *EuSAUR62* overexpressed (*OE-EuSAUR62*) plants. Plants were cultured in a glasshouse. A nursery bed was prepared to raise rice seedlings, and irrigation water was applied by sprinkling. Healthy seedlings were selected and transplanted to pots (one seedling in each pot) with 2 cm of standing water in April. During culture, the plants were supplied with compound fertilizer when needed. The image data of plants were recorded every 2 days after transplanting, and rice leaves were collected and stored at –80°C for histological analysis on days 10th and 60th after transplanting. Grains from each plant were harvested and kept separate for phenotypic analysis at maturity.

### Metabolite analysis

Metabolites of mature Euryale seeds were detected by LC-MS as previously described (Wu et al., 2013; Wang et al., 2015). Briefly, 4 g power of the sample was extracted in 40 ml extraction buffer (methanol: double distilled water = 4:1, v/v) by ultrasonication. Then, the samples were centrifuged at 10,000 rpm for 5 min; the upper phase was dried under a stream of nitrogen gas and redissolved with 1 ml chromatographic methanol. Each sample solution was injected into the reverse-phase C_18_ column (250 mm × 4.6 mm, 5 μm, Agilent, United States) for LC-MS analysis (Ultra-Fast Liquid Chromatography, Shimadzu, Japan; Triple TOFTM 5600, AB Sciex, United States). The qualitative analysis and the resolution of the chromatogram of the target components were carried out using the MS-DIAL software 3.98. The differences in metabolites were calculated using one-way ANOVA and *post hoc* statistical analysis.

### Histological analysis

For section observation, fruit and leaf samples were cut into 30 μm sections with a microtome RM-2235 (Leica Microsystems, Germany) following a series of dehydration and infiltration steps. Sections were then stained with 1% safranin and 1% Fast Green and imaged under a Carl Zeiss Axio Scope fluorescence microscope (ZEISS Gottingen, Germany). The cell lengths and widths were measured using the ImageJ 1.51 software.

For scanning electron microscopy (SEM) observations, glumes of rice were separately collected from the *OE-EuSAUR62* and WT lines at the heading stage. After imaging the whole glume using a stereomicroscope (V20, ZEISS), samples were cut into small pieces and were gold plated followed by observation using a scanning electron microscope (LEO1530VP, ZEISS). The lengths and widths of the outer glume cells were measured using the ImageJ 1.51 software.

### Phylogenetic analysis

Arabidopsis and rice SAUR protein sequences were downloaded from the Arabidopsis Information Resource^1^ and the China Rice Data Center^2^ websites, respectively. The phylogenetic tree was constructed using the MEGA 5.0 software *via* the neighbor-joining method.

### RNA extraction and qRT-PCR

Total RNA was extracted from various tissues using TRIzol reagent (T9424, Sigma, United States) and 1 μg was used for cDNA synthesis with the PrimeScript RT Master Mix Kit (RR036A, TaKaRa, China). qRT-PCR was performed using a QuanStudio 3 instrument (Applied Biosystems, United States), and the β-actin was used as an internal control for normalization as described previously (Huang et al., 2020; Jing et al., 2021). At least three biological and three technical replicates were employed for all samples, and the expression data were generated using the comparative Ct (ΔΔCt) method after confirming a single product by disassociation curves.

### Subcellular localization assay

The pCambia1300 vector was used for subcellular localization assays. The *p35S:EuSAUR62-GFP*, *p35S:OsPIN9-GFP*, *p35S:EubZIP54-GFP*, and *p35S:EubZIP55-GFP* vectors consisted of the full-length CDS of relative genes fused with the green fluorescent protein (GFP) reporter gene and was driven by the 35S promoter. The vectors were transformed into tobacco (*Nicotiana tabacum* L.) leaves, followed by overnight incubation staying in the dark, as previously described (Li et al., 2021). GFP signals were visualized using a laser confocal microscope (C3-ER, Nikon, Japan) after culturing under normal conditions for 3 days.

### Generation of transgenic plants

A 429 bp cDNA without the stop codon was amplified from Euryale using the primers *OE-EuSAUR62-F* and *OE-EuSAUR62-R* (Supplementary Table 2) and inserted into the binary vector pCambia1300 *via Kpn*I and *Xba*I sites. The *p35S:EuSAUR62-GFP* and *p35S:empty-GFP* plasmids were introduced into the *Agrobacterium tumefaciens* strain EHA105. The constructs were transferred into the wild rice by infecting calli, as previously reported (Dang et al., 2020).

For promoter activity analysis, a 1616 bp genomic fragment was amplified using *EuSAUR62-p-F* and *EuSAUR62-p-R* (Supplementary Table 2) and replaced the original 35S promoter of pCambia1301 *via Pst*I and *Nco*I sites. The constructed plasmid was cloned into GV3101, and then the positive clones were injected into the leaves to construct transient expression tobacco.

### Western blot

Rice leaves were lysed in protein extraction buffer containing 10 mM Tris–HCl (pH = 8), 1 mM Phenylmethylsulfonyl fluoride, 60 mM β-glycerophosphate, and 2% SDS. Samples were then rapidly homogenized and western blots were performed. Anti-GFP (66002-1-lg, Proteintech Group, China) and anti-β-actin (6008-1-lg, Proteintech Group, China) were used as primary antibodies. Horseradish peroxidase (HRP)-conjugated goat IgG (H + L) (SA00001-1, SA00001-2, Proteintech Group, China) was used as the secondary antibody. Blots were visualized using the SuperSignal West Pico Chemiluminescent Substrate (Thermo Fisher Scientific Inc.). I WT was used as a negative control. All experiments were performed in triplicate.

### Immunofluorescence staining

Immunofluorescence staining for free-IAA was carried out on sections of the rice ovary as described previously (Nishimura and Koshiba, 2019; Huang et al., 2020), with some modifications: 80 μm sections were dehydrated for 5 min in ascending and descending 25, 50, 75, and 100% methanol solutions, incubated in 0.1% Sudan black b (dissolved in 70% methanol) for 10 min to reduce the autofluorescence, washed three times for 5 min in 50% methanol. Sections were incubated in detergent solution (DMSO: Triton X-100: PBS = 10: 3: 87, v: v: v) for 30 min followed by washing three times. Then, sections were blocked with 5% (w/v) bovine serum albumin in PBS for 1 h, incubated with anti-IAA antibody (1:200, AS09421, Agrisera, Sweden) overnight. After washing three times with 0.1% (v/v) Tween 20 in PBS buffer for 5 min, sections were incubated with Alexa Fluor 488 [1:200, conjugated Goat Anti-Rabbit IgG (H + L), SA00006-2, Proteintech Group, United States] for 1 h in dark. Fluorescence signals (green) were imaged on a Carl Zeiss Axio Scope fluorescence microscope. PBS was added instead of anti-IAA antibodies as a negative control. All experiments were performed in triplicate.

### Bimolecular fluorescence complementation assay

For the BiFC assay, the *OsPIN9* CDS was inserted into the pFGC-YC155 vector to generate the OsPIN9-cYFP. The *EuSAUR62* CDSs were inserted into the pFGC-YN173 vector to generate the EuSAUR62-nYFP. After transformation into Agrobacterium strain GV3101, all fusion proteins were co-expressed in tobacco leaves. After 3 days, the yellow fluorescence protein (YFP) signal was detected at a range of 530-630 under a laser scanning confocal microscope (SP8; Leica, Wetzlar, Germany).

### Co-immunoprecipitation assay

Co-immunoprecipitation assay was used to test protein interactions as described previously with some modifications (Chen et al., 2021). The *p35S:EuSAUR62-Flag* and *p35S:OsPIN9-GFP* vectors were constructed and co-transfected into tobacco leaves. After extraction of total protein, the liquid supernatant was mixed with rProtein A/G MagPoly beads (SM01510, SMART Lifesciences, China) overnight at 4°C on a rotating wheel after binding anti-GFP antibody to the beads. Immunocomplexes were washed six times in IP buffer, and eluted by boiling with 40 μl of SDS sample buffer. Then, the immunocomplexes was assessed by western blot with anti-Flag (M20008XS, Abmart, China) and anti-GPF antibodies.

### Membrance yeast two-hybrid assay

The DUAL membrane system was used to assay the interactions between the EuSAUR62 and OsPIN9. Full-length *EuSAUR62* and *OsPIN9* were cloned into pBT3–SUC and pPR3–N, respectively. Transformation of NMY51 yeast strain was performed using standard procedures as described previously (Chen et al., 2021). The positive strains were transferred onto minimal SD agar base plates supplemented with DO Suppleme–t – Trp, Leu, and His (SD-T-L-H) and increasing 3-Amino-1,2,4-triazole (3-AT) supplement. Plates were checked for interactions after 2–3 days of incubation at 30°C.

### Genome walking

The promoter of *EuSAUR62* was obtained using a genome walking kit according to the manufacturer’s instructions (TaKaRa, Dalian, China). Random primers (AP1–AP4) were provided by the genome walking kit, and the specific primers were designed based on the known sequences (first round EuSAUR-r1; second round EuSAUR-r2; third round EuSAUR-r3, Supplementary Table 2). Specific PCR products were purified using the DNA Gel Extraction Kit (Axygen, United States), cloned into the pMD-18 vector system, and then sequenced.

### β-glucuronidase staining

After growth under normal conditions for 3 days, the transgenic tobacco leaves were overnight stained with β-glucuronidase (GUS) staining buffer {50 mM sodium phosphate buffer, pH 7.2, 10 mM EDTA, 1 mM K3[Fe(CN)6], 0.5 mM K4[Fe(CN)6]^⋅^3H_2_O, 0.1% (v/v) Triton X-100, 1 mg/ml X-Gluc} at 37°C. The samples were then washed with 70% ethanol to remove the chlorophyll. Images were captured using a stereomicroscope (V20, ZEISS).

### DNA-pulldown assay

A ∼1600 bp *EuSAUR62* promoter (*EuSAUR62pro*) region was amplified using the biotinylated primers. This probe was then added to the fruit lysates of the HL and WT, respectively, and incubated at room temperature. Probe-protein complexes were precipitated with DynaBeads (Beaver Biosciences Inc., China) for 1 h. Beads were washed two times with nucleic acid incubation buffer and eluted with 1% sodium deoxycholate dissolved in 100 mM Tris-HCl buffer (pH = 8.5). The products were incubated at 95°C for 10 min, separated by SDS-PAGE, and then stained with silver. The gel bands were excised and digested with trypsin (0.6 mg), and the tryptic peptides were subjected to LC-MS/MS (EKspert*™* nanoLC; AB Sciex TripleTOF 5600-plus) analysis. Sequence and peptide data were aligned against the predicted proteome of *O. sativa* L. and *A. thaliana* using the ProteinPilot software.

### Yeast one-hybrid assay

The yeast one-hybrid (Y1H) was used to test direct interactions between EubZIPs and *EuSAUR62pro* as previously described (Xi et al., 2019). The CDS of *EubZIP54* was inserted into the pGADT7 vector at the *Bam*HI and *Xho*I sites, and *EubZIP55* was inserted into the pGADT7 vector at the same sites, whereas the *EuSAUR62pro* was inserted into the pHIS2.1 vector at the *Mlu*I and *Sac*II sites. The resultant vectors were inserted into yeast strain Y187 cells, according to the manufacturers instructions (Clontech). Transformed yeast cells were grown on SD-T-L medium at 30°C for 3–5 days. The positive clones were transferred to SD-T-L-H but supplemented with increasing 3-AT concentration at 30°C for 3–5 days.

### Dual luciferase reporter assay

To study the transcriptional activity of EubZIP54/55 on *EuSAUR62*, we performed a Dual luciferase reporter assay (LUC). The *p35S:EubZIP54-GFP* and *p35S:EubZIP55-GFP* were used as effector vectors. The *p35S:empty-GFP* was an empty effector vector. The *EuSAUR62pro* was inserted into the reporter vector pGreen II 0800-LUC. The effector vectors and reporter vectors were co-transfected into tobacco leaves. The reaction was performed using the Dual Luciferase Reporter Assay Kit (DL101-01, Vazyme, China) and luciferase activities were measured by the microplate reader (INFINITE2000, TECAN, Switzerland). All experiments were performed in triplicate.

### Seedlings treatment and hook curvature measurement

Euryale seeds were cultured in water for sprouting in a plant growth chamber at a constant temperature (∼20°C) and a cycle of 14-h light/10-h dark. Two-week-old seedlings of HL and WT were randomly divided into four groups: (1) HL seedlings with constant light treatment for 7 days; (2) the WT seedlings with constant light treatment for 7 days; (3) HL seedlings cultured in the dark for 7 days; (4) WT seedlings cultured in the dark for 7 days. During treatment, images of each seedling were captured using a digital single lens reflex camera (Canon, Tokyo) every day. Hook angles of individual seedlings were measured digitally. All experiments were performed in triplicate.

### Data analysis

All data were expressed as the mean ± SD. Differences between two groups were analyzed using the *t*-test, while differences among groups were analyzed using one-way ANOVA, and *p* < 0.05 was represented as a statistically significant difference. Statistical analysis and frequency distribution analysis was carried out by GraphPad Prism 6.0 software (GraphPad Software, Inc.).

## Results

### The cell elongation and ovary enlargement in hybrid Euryale lines were promoted by a high content of indole-3-acetic acid

**FIGURE 1:**
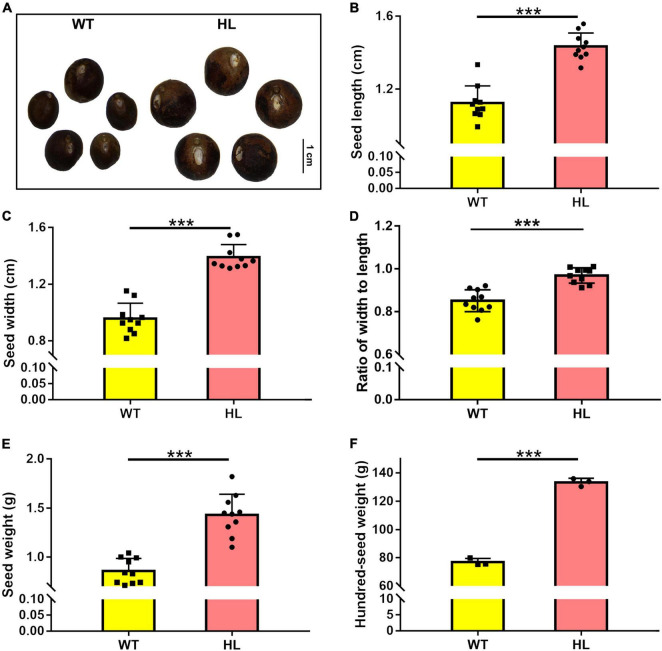
Seed comparison of North Gordon Euryale (WT) and Hybrid Euryale (HL). After harvest in September (20th week), Phenotype **(A)**, length **(B)**, width **(C)**, ratio of width to length **(D)**, weight **(E)**, and hundred-seed weight **(F)** of fresh seeds of the WT and HL were detected. Bar = 1 cm. Data are mean ± SD. Two-tail Student’s *t*-test was performed between the WT and HL (*p < 0.05; **p < 0.01; ***p < 0.001).

In September, fully mature seeds were collected for yield trait analysis. Compared to the WT, HL elevated yield traits of seeds in both size and weight (Figures 1A–E). The yield advantage of HL was also revealed by hundred-seed weight (Figure 1F), and the average yield (WT = 3500 kg/ha and HL = 4,875 kg/ha) in experimental plots. The growth of fruits in the two types showed that, compared to the difference in fruit numbers of each plant between these two varieties, HL obviously produces larger fruits since the 10th week after sowing (Supplementary Figure 1). The seed of HL is obviously rounder than that of WT (Figure 1D), leading us to speculate that there may be differences in fruit histology or seed metabolites between HL and WT.

**FIGURE 2:**
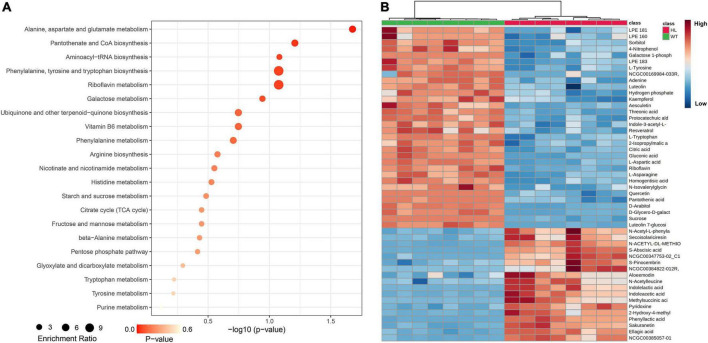
Metabolic profiling analysis of the WT and HL. **(A)** KEGG classification of significantly different contents in dry mature seeds (harvested in the 20th week) between the WT and HL. Each point represents the degree of enrichment of the KEGG entry, and the size of each point indicates the number of different metabolites enriched in the KEGG pathway. **(B)** Heat map of metabolites with significantly different contents. Every square block indicates a metabolite, and significantly up-regulated and down-regulated metabolites are displayed in red and blue, respectively. HL, hybrid Euryale lines; WT, wild type (North Gordon Euryale).

To investigate the difference in metabolites in seeds of WT and HL, we performed untargeted metabolomics of both varieties using liquid chromatography-mass spectrometry (LC-MS) detection with negative ion mode. We detected 94 metabolites in total in mature seeds harvested at the 20th week from the two types (Table 1), and 50 different components between HL and WT, including phenolic acids, amino acids, flavonoids, organic acids, and their derivatives, are shown in Figure 2B. KEGG analysis revealed that these different metabolites are enriched in various pathways, such as metabolic pathways in the biosynthesis of alanine, aspartate, glutamate, phenylalanine, tyrosine, and tryptophan biosynthesis, and riboflavin metabolism (Figure 2A). A clear separation between HL and WT was observed using principal component analysis (PCA) and orthogonal projections to latent structures analysis (OPLS-DA), suggesting remarkably different metabolites in the seeds of two lines (Supplementary Figure 2). Among these metabolites, the indole-3-acetic acid (Figure 2B) and tryptophan metabolism pathway (Figure 2A) attracted our attention. IAA was significantly enriched in the seed of HL, indicating that IAA management between HL and WT is different during fruit growth. According to our previous study, there is no difference in IAA concentration between HL and WT fruits at the same stage, but the IAA distribution is different (Huang et al., 2020). Specifically, the WT allocates free-IAA to its pricky epicarp more frequently, whereas the fluorescence signal in fruits of HL is enriched in the ovary and stamen, indicating a potential role for IAA hemostasis in regulating fruit development.

**TABLE 1:** Metabolites of both WT and HL seeds.

Num.	Metabolite name	Formula	RT (min)	Adduct type
1	Ala-Ala	C_6_H_12_N_2_O_3_	1.995	[M-H]^–^
2	Cytidine	C_9_H_13_N_3_O_5_	2.017	[M-H] ^–^
3	Adenine	C_5_H_5_N_5_	2.134	[M-H] ^–^
4	Pyridoxine	C_8_H_11_NO_3_	2.159	[M-H] ^–^
5	Serine	C_3_H_7_NO_3_	2.177	[M-H] ^–^
6	L-Asparagine	C_4_H_8_N_2_O_3_	2.186	[M-H] ^–^
7	Aspartic acid	C_4_H_7_NO_4_	2.305	[M-H] ^–^
8	Perseitol	C_7_H_16_O_7_	2.368	[M-H] ^–^
9	9-Fluorenone	C_13_H_8_O	2.439	[M-H] ^–^
10	Sorbitol	C_6_H_14_O_6_	2.439	[M-H] ^–^
11	Arabitol(D)	C_5_H_12_O_5_	2.468	[M-H] ^–^
12	Sucrose	C_12_H_22_O_11_	2.485	[M-H] ^–^
13	Gluconic acid	C_6_H_12_O_7_	2.566	[M-H] ^–^
14	Threonic acid	C_4_H_8_O_5_	2.673	[M-H] ^–^
15	2-Ketogluconic acid	C_12_H_24_O_9_	2.676	[M-H] ^–^
16	Uridine	C_9_H_12_N_2_O_6_	2.905	[M-H] ^–^
17	Guanosine	C_10_H_13_N_5_O_5_	3.093	[M-H] ^–^
18	Ortophosphate	H_3_O_4_P	3.539	[M-H] ^–^
19	Hypoxanthine	C_5_H_4_N_4_O	3.676	[M-H] ^–^
20	Tyrosine	C_9_H_11_NO_3_	3.734	[M-H] ^–^
21	Glucose-1-phosphate	C_6_H_13_O_9_P	4.023	[M-H] ^–^
22	Citrate	C_6_H_8_O_7_	4.275	[M-H] ^–^
23	Inosine	C_10_H_12_N_4_O_5_	4.625	[M-H] ^–^
24	D-Pantothenic acid	C_9_H_17_NO_5_	6.105	[M-H] ^–^
25	Gallic acid	C_7_H_6_O_5_	6.556	[M-H] ^–^
26	Methylsuccinic acid	C_5_H_8_O_4_	6.711	[M-H] ^–^
27	Tryptophan	C_11_H_12_N_2_O_2_	6.716	[M-H] ^–^
28	Homogentisic acid	C_8_H_8_O_4_	7.358	[M-H] ^–^
29	(2S,3S)-2-(3,4,5-trihydroxyphenyl)-3,4-dihydro-2H-chromene-3,5,7-triol	C_15_H_14_O_7_	8.533	[M-H] ^–^
30	*N*-Acetyl-DL-methionine	C_7_H_13_NO_3_S	8.973	[M-H] ^–^
31	2,3-Dihydroxybenzoic acid	C_7_H_6_O_4_	9.332	[M-H] ^–^
32	Epigallocatechin	C_15_H_14_O_7_	9.708	[M-H] ^–^
33	2-Isopropylmalic acid	C_7_H_12_O_5_	9.733	[M-H] ^–^
34	*N*-Isovalerylglycine	C_7_H_13_NO_3_	9.897	[M-H] ^–^
35	(–)-Riboflavin	C_17_H_20_N_4_O_6_	10.018	[M-H] ^–^
36	Protocatechuic aldehyde	C_7_H_6_O_3_	10.728	[M-H] ^–^
37	Methylgallate	C_8_H_8_O_5_	10.811	[M-H] ^–^
38	NCGC00385057-01!	C_27_H_22_O_18_	11.086	[M-H] ^–^
39	Catechol	C_6_H_6_O_2_	11.164	[M-H] ^–^
40	*N*-Acetylleucine	C_8_H_15_NO_3_	11.571	[M-H] ^–^
41	Catechin	C_15_H_14_O_6_	11.763	[M-H] ^–^
42	6,7-Dihydroxycoumarin	C_9_H_6_O_4_	11.942	[M-H] ^–^
43	NCGC00347753-02_C19H18N4O7_9H-Purin-6-ol, 9-[1-(3-carboxyphenyl)-2,3-dideoxyheptodialdo-7,4-furanosyl]-	C_19_H_18_N_4_O_7_	11.942	[M-H] ^–^
44	NCGC00384822-01![(2R,3S,4S,5R,6S)-3,4-dihydroxy-5,6-bis[(3,4,5-trihydroxybenzoyl)oxy]oxan-2-yl]methyl 3,4,5-trihydroxybenzoate	C_27_H_24_O_18_	12.057	[M-H] ^–^
45	2-Hydroxy-4-methylpentanoic acid	C_6_H_12_O_3_	12.126	[M-H] ^–^
46	Epigallocatechin-3-gallate	C_22_H_18_O_11_	13.232	[M-H] ^–^
47	Myricetin-3-Galactoside	C_21_H_20_O_13_	13.576	[M-H] ^–^
48	*N*-acetylphenylalanine	C_11_H_13_NO_3_	13.981	[M-H] ^–^
49	3-Phenyllactic acid	C_9_H_10_O_3_	14.086	[M-H] ^–^
50	Indole-3-acetyl-L-glutamic acid	C_15_H_16_N_2_O_5_	14.443	[M-H] ^–^
51	Luteolin-7-*O*-glucoside	C_21_H_20_O_11_	14.604	[M-H] ^–^
52	Hyperoside	C_21_H_20_O_12_	15.089	[M-H] ^–^
53	Epicatechin gallate	C_22_H_18_O_10_	15.336	[M-H] ^–^
54	Indolelactic acid	C_11_H_11_NO_3_	15.43	[M-H] ^–^
55	Ellagic Acid	C_14_H_6_O_8_	15.576	[M-H] ^–^
56	Syringetin-3-*O*-glucoside	C_23_H_24_O_13_	15.816	[M-H] ^–^
57	Kaempferol-3-*O*-glucoside	C_21_H_20_O_11_	16.238	[M-H] ^–^
58	Taxifolin	C_15_H_12_O_7_	16.439	[M-H] ^–^
59	3-(2-Hydroxyphenyl)propanoic acid	C_9_H_10_O_3_	16.448	[M-H] ^–^
60	Secoisolariciresinol	C_20_H_26_O_6_	16.682	[M-H] ^–^
61	NCGC00169984-03!(3R,5R)-3,Iis[[(E)-3-(3,4-dihydroxyphenyl)prop-2-enoyl]oxy]-1,4-dihydroxycyclohexane-1-carboxylic acid	C_25_H_24_O_12_	16.983	[M-H] ^–^
62	Indole-3-carboxaldehyde	C_9_H_7_NO	17.684	[M-H] ^–^
63	Indole-3-acetic acid	C_10_H_9_NO_2_	18.021	[M-H] ^–^
64	Abscisic acid	C_15_H_20_O_4_	18.818	[M-H] ^–^
65	Sebacic acid	C_10_H_18_O_4_	19.407	[M-H] ^–^
66	Tricetin	C_15_H_10_O_7_	19.495	[M-H] ^–^
67	Myricetin	C_15_H_10_O_8_	19.559	[M-H] ^–^
68	Eriodictyol	C_15_H_12_O_6_	20.58	[M-H] ^–^
69	4-Nitrophenol	C_6_H_5_NO_3_	20.731	[M-H] ^–^
70	*Trans*-Resveratrol	C_14_H_12_O_3_	20.737	[M-H] ^–^
71	Luteolin	C_15_H_10_O_6_	21.967	[M-H] ^–^
72	LPE 12:0	C_17_H_36_NO_7_P	22.044	[M-H] ^–^
73	Quercetin	C_15_H_10_O_7_	22.306	[M-H] ^–^
74	Phloretin	C_15_H_14_O_5_	22.484	[M-H] ^–^
75	5,7-dihydroxy-2-(4-hydroxy-3-methoxyphenyl)-2,3-dihydrochromen-4-one	C_16_H_14_O_6_	22.542	[M-H] ^–^
76	LPE 13:0	C_18_H_38_NO_7_P	23.221	[M-H] ^–^
77	Aloe-emodin	C_15_H_10_O_5_	24.29	[M-H] ^–^
78	LPE 15:1	C_20_H_40_NO_7_P	24.366	[M-H] ^–^
79	Kaempferol	C_15_H_10_O_6_	24.882	[M-H] ^–^
80	LPE 18:3	C_23_H_42_NO_7_P	24.969	[M-H] ^–^
81	LPE 16:1	C_21_H_42_NO_7_P	25.132	[M-H] ^–^
82	LPE 15:0	C_20_H_42_NO_7_P	25.328	[M-H] ^–^
83	Sakuranetin	C_16_H_14_O_5_	25.49	[M-H] ^–^
84	Pinocembrin	C_15_H_12_O_4_	26.071	[M-H] ^–^
85	LPE 18:2	C_23_H_44_NO_7_P	26.184	[M-H] ^–^
86	LPC 18:2	C_26_H_50_NO_7_P	26.282	[M + FA-H] ^–^
87	Chrysin	C_15_H_10_O_4_	27.05	[M-H] ^–^
88	LPE 16:0	C_21_H_44_NO_7_P	27.189	[M-H] ^–^
89	LPC 16:0	C_24_H_50_NO_7_P	27.319	[M + FA-H] ^–^
90	LPE 18:1	C_23_H_46_NO_7_P	27.786	[M-H] ^–^
91	LPC 18:1	C_26_H_52_NO_7_P	27.912	[M + FA-H] ^–^
92	LPE 17:0	C_22_H_46_NO_7_P	28.568	[M-H] ^–^
93	Beta-Hydroxymyristic acid	C_14_H_28_O_3_	30.224	[M-H] ^–^
94	Octadecanedioic acid	C_18_H_34_O_4_	32.584	[M-H] ^–^

**FIGURE 3:**
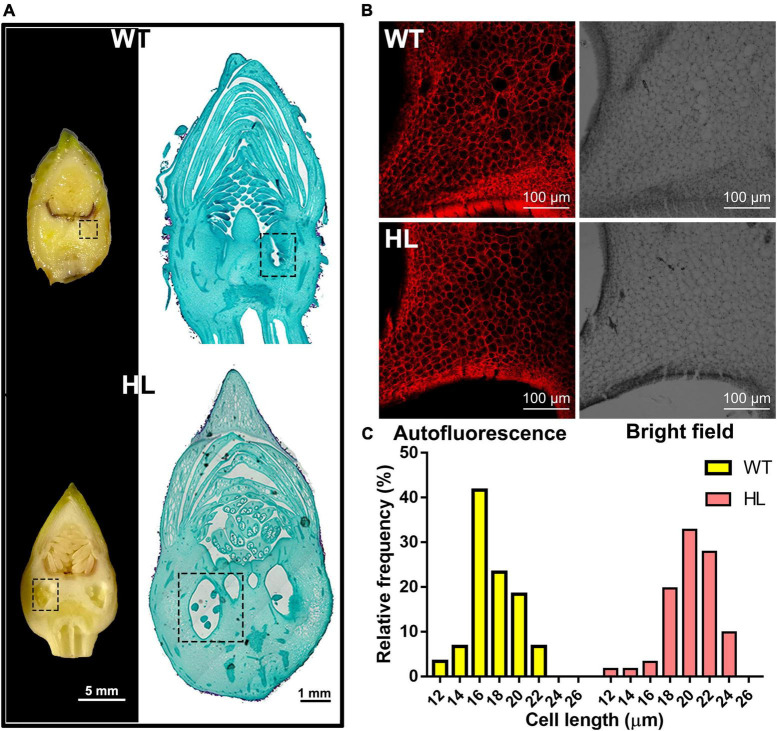
Histological analysis of the ovary of WT and HL fruits. **(A)** Longitudinal sections (left, Scale bar = 5 mm) and representative paraffin sections of the reproductive organizations of 12-week-old WT and HL (right, Scale bar = 1 mm). The ovary, denoted with the dotted box, was larger in HL fruits when compared with WT at the same developmental stage. **(B)** Cells around the ovary of WT and HL were observed using a fluorescent microscope. Images including bright field and red autofluorescence are shown. Scale bar = 100 μm. **(C)** Cell length of WT and HL were evaluated using six 12-week-old fruits per line. Statistical count was shown as the frequency distribution of the cell length. HL, hybrid Euryale lines; WT, wild type (North Gordon Euryale).

Then, we performed microscopic analysis on the 12-week-old fruits to investigate the difference in fruit histology between HL and WT. The longitudinal sections of both types of fruits are shown in Figure 3A, compared to the WT, the larger ovaries were observed in HL. We then measured the length of cells around the ovary by observing their autofluorescence (red, Figure 3B). As shown in Figure 3C, the cell length in HL and WT continuously varied from 18 to 24 μm and 16 to 20 μm, respectively. Therefore, we concluded that HL produces larger ovaries by distributing its auxin around the ovaries, thereby promoting the elongation of cells around them, which contributes to larger and rounder seeds.

### *EuSAUR62* improved yield traits by increasing cell length in rice

**FIGURE 4:**
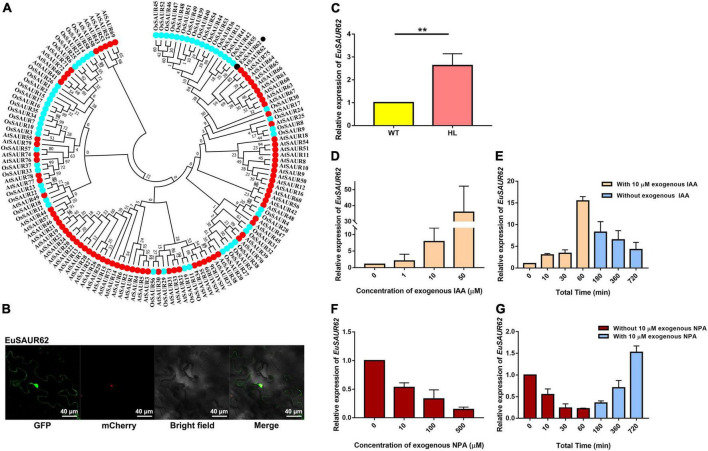
Classification and auxin response of *EuSAUR62*. **(A)** Phylogenetic relationships of EuSAUR62 and SAURs from *Arabidopsis thaliana* and *Oryza sativa*. SAURs from Arabidopsis and rice are displayed in red and cyan, respectively. EuSAUR62 (black) is next to AtSAUR62 and AtSAUR75. **(B)** The subcellular localization of EuSAUR62 in tobacco leaves. EuSAUR62 was fused to GFP and transfected into tobacco leaves. A merged image including bright field, H2B-mCherry and GFP fluorescence was observed using a confocal laser scanning microscope (Scale bar = 40 μm). **(C)** Relative expression of *EuSAUR62* in the fruits of WT and HL was detected using qRT-PCR. qRT-PCR analyses of *EuSAUR62* expression in 2-week-old WT seedlings after treating with different doses of exogenous IAA (0, 1, 10, 50 μM) for 1 h **(D)** or with 10 μM exogenous IAA from 0 to 60 min, followed by replacing the IAA solution with pure water **(E)**. The time points of IAA treatment are shown in orange columns, whereas water culturing is expressed in blue columns. Euryale *Actin* was used as an internal control. Relative expression of *EuSAUR62* in WT seedlings following 1 h treatment of 0, 10, 100, or 500 μM exogenous NPA **(F)**, an auxin transport inhibitor, or with 10 μM exogenous NPA treatment for 1 h before water culturing **(G)**. The time points of NPA treatment are shown in crimson columns, whereas water culturing is expressed in blue columns. Two-tail Student’s *t*-test was used for statistical analysis; Data are mean ± SD. *p < 0.05. HL, hybrid Euryale lines; IAA, Indole-3-acetic acid; NPA, *N*-1-naphthylphthalamic acid; WT, wild type (North Gordon Euryale).

In a previous study, we found that some *SAURs* act as positive mediators of the auxin transduction pathway which might mediate the location of IAA (Huang et al., 2020). In this study, *EuSAUR62*, the most differently expressed in HL and WT, was selected to analyze its potential role in auxin regulation. Phylogenetic analysis revealed that EuSAUR62 was highly homologous to AtSAUR62 and AtSAUR75 (Figure 4A), which have been reported to promote cell elongation in Arabidopsis (He et al., 2018). Subcellular location assay showed that EuSAUR62 is located at the plasma membrane and nucleus (Figure 4B and Supplementary Figure 3A). As shown in Figure 4C, the expression level of *EuSAUR62* in HL was increased about 2.5-fold compared to the WT. We speculated that it is the localization of auxin that causes the up-regulation of *EuSAUR62* expression in HL, as its homologous gene belongs to an auxin-response *SAUR*-clade (van Mourik et al., 2017; Kathare et al., 2018). To verify the response manner of the *EuSAUR62* gene, we analyzed the expression of *EuSAUR62* in seedlings under exogenous IAA or NPA treatment as reported previously (Kant et al., 2009). It was found that *EuSAUR62* is up-regulated from 0 to 60 min after 10 μm IAA treatment (Figure 4E), while is down-regulated by application of 10 μm exogenous NPA (Figure 4G), an inhibitor of auxin transport, and the expression in both treatment groups gradually return to normal level after discontinuing treatment. As shown in Figures 4D,F, exogenous IAA and NPA dynamically increased and decreased the expression level of *EuSAUR62*, respectively, and both showed dose-response relationships. In addition, an elevated expression of *EuSAUR62* after treatment with 1-naphthaleneacetic acid (NAA) suggested that *EuSAUR62* can positively respond to different types of auxins (Supplementary Figure 3B). Our results implied that *EuSAUR62* responds to alteration of auxin concentration in a rapid manner.

**FIGURE 5:**
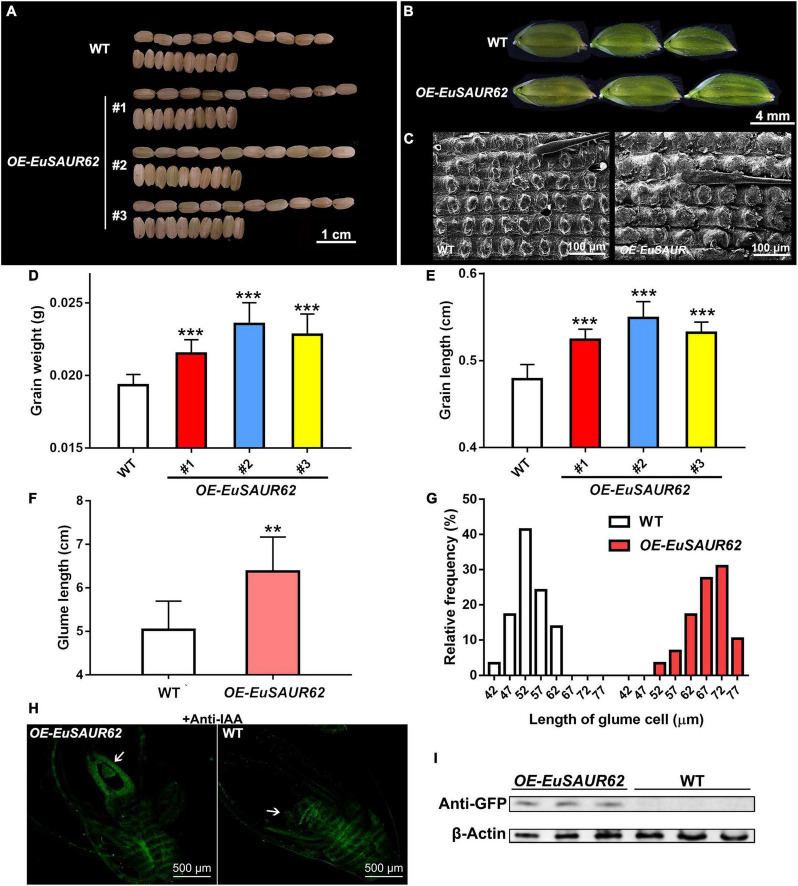
Grain size and histology comparison of the WT and *OE-EuSAUR62* rice. After harvest, **(A)** morphologies of the mature grains produced by *OE-EuSAUR62* and WT were observed. The grains generated by the *OE-EuSAUR62* were significantly longer than that of WT. Scale bar = 1 cm. Histological analysis of the glumes **(B)** and outer glume cells **(C)** at heading stage. The glumes of WT and transformant were observed using a stereo microscope (Scale bar = 4 mm). The outer glume cells were observed under a scanning electron microscope (Scale bar = 100 μm). After harvest, 10 grains per line were collected randomly for size and weight measurement. *OE-EuSAUR62* significantly improved the grains in weight **(D)** and length **(E)** compared with the WT. The glume length of *OE-EuSAUR62* rice was longer than the WT **(F)**. Length of the outer glume cells was longer in *OE-EuSAUR62* rice compared to the WT **(G)**. Statistical count is shown as the frequency distribution of the cell length. **(H)** Localization of IAA in the rice ovary (white arrow) of WT and *OE-EuSAUR62* at the flowering stage (Scale bar = 500 μm). **(I)** The expression of EuSAUR62 was detected using western blot analysis with an anti-GFP antibody. The β-actin was used as an internal control. The negative controls are represented in the Supplementary material. Data are mean ± SD. One-way ANOVA and *post hoc* statistical analysis was performed (**p < 0.01, ***p < 0.001). *OE-EuSAUR62*, *EuSAUR62* overexpression lines; WT, wild type rice.

Since it is difficult to perform the genetic transformation in Euryale, we used rice (*Oryza sativa* L. ssp. *japonica* cv. Nipponbare) as the recipient material for transformation to explore whether alterations in grain weight, grain size, and other phenotypes are associated with *EuSAUR62*. The phenotypes of grains are shown in Figure 5A, grains from *EuSAUR62* gain-of-function lines were more than 12% heavier and 10% longer (*p* < 0.001, Figures 5D,E), whereas there was no difference in grain width between the two lines (*p* > 0.05, Supplementary Figure 4B) compared to the WT, revealing that *EuSAUR62* increases length and weight of grains. F2 populations of *EuSAUR62* fit the expected 3:1 ratio (χ^2^ = 0.0525, df = 1, *p* = 0.8187) for a single segregating transgene. The EuSAUR62 protein was detected in larger grains by immunodetection using a GFP antibody, further supporting our results (Supplementary Figure 4A).

In addition to the increased grain size and weight observed in the *OE-EuSAUR62*, the *OE-EuSAUR62* also produced larger glumes (Figure 5B). Glumes in *OE-EuSAUR62* lines had an average length of approximately 6.38 mm, which was significantly longer than that of the WT (*p* < 0.01, Figure 5F), whereas no difference was observed in the glume width between the two lines (*p* > 0.05, Supplementary Figure 4C). Further histological analysis using SEM revealed an increase in the length of outer glume cells of *OE-EuSAUR62* lines (Figures 5C,G), indicating that *EuSAUR62* regulates grain size by modulating the longitudinal cell length in the lemma. Thus, our results provided evidence that *EuSAUR62* positively regulates cell length and glume size.

Moreover, we detected other altered phenotypes in *OE-EuSAUR62* plants, including plant height and leaf angle. For the plant height, we found that, on day 10, after transplantation, young leaves of *OE-EuSAUR62* lines grow much faster than WT (Supplementary Figures 5A,C). We also observed a significantly enlarged leaf angle in the bottom leaves of *OE-EuSAUR62* plants on the 60th day (Supplementary Figures 5E,G). Further histological observation of leaf parenchyma cells and pulvinus cells by autofluorescence revealed that most parenchyma cells in *OE-EuSAUR62* lines and WT are distributed at 44–74 and 34–64 μm, respectively (Supplementary Figures 5B,D), and there are more pulvinus cells above 64 μm in the *OE-EuSAUR62* lines than in the WT (Supplementary Figures 5F,H). Collectively, these results suggested that *EuSAUR62* promotes the elongation of both parenchyma and pulvinus cells, strongly confirming its role for *EuSAUR62* in promoting cell length.

As previously reported, IAA distribution is significantly different in two Euryale (Huang et al., 2020). We speculated that *EuSAUR62* mediates IAA distribution during fruit development. Thus, to determine whether *EuSAUR62* regulates the distribution of IAA in rice, we performed an immunofluorescence assay with an anti-IAA antibody on reproductive tissue sections at the flowering phase. After excluding the background fluorescence in the negative control, which was incubated with 1% BSA instead of the primary antibody (Supplementary Figure 4D), we found that *OE-EuSAUR62* lines allocate free-IAA to the ovary more frequently compared to the WT (Figure 5H), indicating that *EuSAUR62* regulates the location of IAA in reproductive tissues. The expressed EuSAUR62 protein was also detected in *OE-EuSAUR62* rice by western blot assay (Figure 5I).

### EuSAUR62 directly interacted with OsPIN9 protein

**FIGURE 6:**
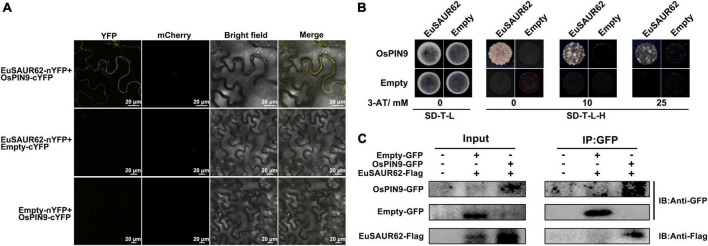
Interaction of EuSAUR62 and OsPIN9. **(A)** EuSAUR62 and OsPIN9 were fused with the corresponding YFP parts for BiFC assay in tobacco leaves. Images including bright field, H2B-mCherry and YFP fluorescence were observed using a laser confocal microscope (Scale bar = 20 μm). **(B)** Membrane yeast two-hybrid assay of the interaction of EuSAUR62 and OsPIN9. Interactions were determined based on cell growth on SD-T-L-H with 3-AT. IAA, Indole-3-acetic acid; *OE-EuSAUR62*, *EuSAUR62* overexpression lines; WT, wild type rice. **(C)** Tobacco leaves were transfected with constructs containing a *p35S:OsPIN9-GFP* and/or *p35S:EuSAUR62-Flag*. Proteins were immunoprecipitated with anti-GFP antibodies and subjected to western blot analysis with anti-Flag antibodies to confirm the presence of the OsPIN9 protein and to anti-GFP gel-blot analysis to reveal the EuSAUR62 partner.

Indole-3-acetic acid homeostasis is controlled by both local IAA biosynthesis and polar IAA transport, but there is no significant change in IAA synthesis genes between the WT and HL at the same growth stage according to our transcriptome data, suggesting that the accumulation of IAA was induced not by local biosynthesis (Supplementary Figure 6). Based on network analysis in the STRING database (the OsSAUR39 was the most homologous to EuSAUR62 in the database), OsPIN9 was screened as a candidate that interacts with the OsSAUR39 protein (Supplementary Figure 7A), further indicating that the accumulation of IAA was mediated by polar transport. Polar IAA transport is controlled by several carrier proteins, such as AUX/LAX, PIN, and ABC transporters (Kamimoto et al., 2012; He et al., 2017). Transient expression of OsPIN9 in tobacco leaves revealed that it is expressed at the plasma membrane (Supplementary Figure 7B). As both EuSAUR62 and OsPIN9 can express at the plasma membrane (Supplementary Figure 3A), we speculated that there could be an interaction between EuSAUR62 and OsPIN9 proteins. To verify our hypothesis, we completed bimolecular fluorescence complementation (BiFC) and membrane yeast two-hybrid assays (mY2H) assays. The physical interaction between EuSAUR62 and OsPIN9 was confirmed in the BiFC assay, as YFP signals could only be detected in samples expressing both EuSAUR62 and OsPIN9 (Figure 6A). We used the DUAL membrane system to analyze the interaction between EuSAUR62 and OsPIN9 on the cellular membrane as previously described (Chen et al., 2021). As shown in Figure 6B, the direct interaction was confirmed in this assay while expressing EuSAUR62 and OsPIN9 in pBT3-SUC and pPR3-N vectors, respectively. However, no interaction was observed when expressing EuSAUR62 and OsPIN9 in the pPR3-N vector and pBT3-SUC vector, respectively, suggesting EuSAUR62 positioned incorrectly by itself in yeast (pBT3-SUC vector expresses an extra signal peptide). Co-immunoprecipitation (Co-IP) assay also revealed the interaction between EuSAUR62 and OsPIN9 *in vivo* (Figure 6C). These results suggested that EuSAUR62 interacts with OsPIN9 and affects the auxin distribution.

### EubZIP55 up-regulated the expression of *EuSAUR62* and mediated indole-3-acetic acid accumulation under light conditions

After confirming the function of *EuSAUR62* in seed size, the question remained as to why *EuSAUR62* is differentially expressed in HL and WT. As the CDS sequences of *EuSAUR62* in two types were proven to be the same, we used genome walking to sequence the promoter (∼1.6 kb) of *EuSAUR62* in HL and WT. However, there was no difference in promoter sequences between the two types (Supplementary Figure 8), suggesting that the different expression of *EuSAUR62* between HL and WT is not attributable to mutations in the gene sequence. Therefore, we focused on transcription factors that regulate *EuSAUR62* expression in Euryale. Then, we used the JASPAR database and DNA-pulldown to screen the transcriptional factors (TFs) that potentially bind to the promoter of *EuSAUR62*. By setting the relative profile score threshold to 100%, we found that its promoter contains specific motifs that can be bound by five types of TFs (Supplementary Table 1). After the products of DNA-pulldown were electrophoresed by SDS-PAGE and silver stained, obviously different protein bands were detected (Supplementary Figure 9A), indicating that different proteins bind to the promoter of *EuSAUR62* (*EuSAUR62pro*) between HL and WT. Next, products of DNA pulldown were identified by LC-MS/MS to identify the different TFs. As shown in Table 2, after filtering low score or uncharacterized proteins, 31 proteins that bind to the *EuSAUR62pro* were detected in HL, and 11 were detected in WT, and among these proteins, the G-box binding factor was identified in both DNA-pulldown and JASPAR assays. In addition, three specific G-box motifs were found in the *EuSAUR62pro* (Supplementary Figure 9B). Thus, we searched the expression profiles of unigenes annotated as G-box binding proteins in transcriptome sequence data (Supplementary Figure 9C), and, finally, two differently expressed *EubZIP54/55* in HL and WT were selected. Phylogenetic analysis revealed that EubZIP54/55 are highly homologous to AtbZIP54/55 in Arabidopsis (Supplementary Figure 9D), belonging to GBF3 and GBF2, respectively (Jakoby et al., 2002).

**TABLE 2:** Proteins from DNA-pulldown were identified by LC-MS/MS.

Group	Names^1^	Coverage (%)	Unused^2^	Reference organism	Unique PepSeq
WT	Histone H2A	19.72	4.29	Arabidopsis	NKGDIGSASQEF, HIQLAVR, AGLQFPVGR
	DNAse I-like superfamily protein	4.71	2.26	Arabidopsis	VSTKWNSLIR, QHEYTRGETK
	Putative far-red impaired response protein	1.13	2	Oryza sativa	KAFNLSGNLLKAK
	Nucleosome assembly protein 1	3.18	2	Oryza sativa	AIGTEIEWYPGK
	Beta-glucosidase-like protein	1.60	2	Arabidopsis	IGEATALEVR
	Alpha/beta-Hydrolases superfamily protein	2.65	2	Arabidopsis	AGFAGDDAPR
	NAC-A/B domain-containing protein	6.90	2	Arabidopsis	SPASDTYVIFGEAK
	Histone H2A	6.51	4	Oryza sativa	INPVLLPK
	Calcium-dependent lipid-binding (CaLB domain) family protein	1.52	2	Arabidopsis	GSFSSVVSDK
	60S ribosomal protein L23A	8.55	2	Oryza sativa	LTPDYDALDVANK
	Phytochrome kinase substrate 1	5.14	2	Arabidopsis	LMEPSNTLNMSINPK
HL	Histone H2B	24.19	6.35	Oryza sativa	LVLPGELAK, QVHPDIGISSK, YNKKPTITSR
	ATP synthase subunit alpha	6.48	6.21	Oryza sativa	VVDALGVPIDGK, TGSIVDVPAGK, AVDSLVPIGR
	Histone H4	31.07	6	Oryza sativa	DNIQGITKPAIR, DAVTYTEHAR, ISGLIYEETR
	ATP synthase subunit beta	5.88	5.22	Oryza sativa	IGLFGGAGVGK, VLNTGSPITVPVGR
	Eukaryotic translation initiation factor 4A-1	6.03	4.74	Arabidopsis	GLDVIQQAQSGTGK, VLITTDLLAR
	Elongation factor 1-alpha	4.70	4.69	Oryza sativa	IGGIGTVPVGR, STTTGHLIYK
	HSP90.1	3.40	4.56	Arabidopsis	GVVDSDDLPLNISR, AVENSPFLER
	HSP70-1	4.30	4.44	Arabidopsis	NAVVTVPAYFNDSQR, TTPSYVAFTDSER
	Fructose-bisphosphate aldolase	7.26	4.44	Oryza sativa	VAPEVIAEYTVR, GILAADESTGTIGK
	Actin-1	7.96	4.26	Oryza sativa	VAPEEHPVLLTEAPLNPK, DAYVGDEAQSKR
	T-complex protein 1 subunit delta	4.49	4	Oryza sativa	GSNQLVIDEAER, LGGTVDDTELIR
	Histone H3	10.65	3.06	Oryza sativa	STELLIR, EIAQDFKTDLR
	NAC-A/B domain-containing protein	13.30	2.98	Arabidopsis	DIELVMTQAGVSR, SPASDTYVIFGEAK
	Sucrose synthase	1.36	2.27	Arabidopsis	IKQQGLNITPR
	Calnexin	2.42	2.26	Oryza sativa	LQNGLECGGAYLK
	Terminal flower 1	7.91	2.24	Arabidopsis	VVGDVLDFFTPTTK
	40S ribosomal protein	2.62	2.23	Oryza sativa	LLILTDPR
	Nucleic acid-binding	8.28	2.21	Arabidopsis	DYQDDKADVILK
	Biotin carboxyl carrier protein of acetyl-CoA carboxylase	4.31	2.11	Arabidopsis	SPGPGEPPFVK
	Mov34/MPN/PAD-1 family protein	5.02	2.04	Arabidopsis	AVAVVVDPIQSVK
	Histone H2A	6.00	2.03	Arabidopsis	AGLQFPVGR
	26S proteasome regulatory particle triple-A ATPase subunit4	3.00	2.01	Oryza sativa	GVLLYGPPGTGK
	ATP synthase	1.93	2.01	Oryza sativa	LAADTPLLTGQR
	Glycin-rich RNA-binding protein	5.56	2	Oryza sativa	DAIEGMNGK
	Ras-related protein	5.31	2	Oryza sativa	LQIWDTAGQER
	L11 domain containing ribosomal protein	5.42	2	Oryza sativa	IGPLGLSPK
	Glycosyl hydrolase family 3 N terminal domain containing protein	1.60	2	Oryza sativa	IGEATALEVR
	G-box binding factor	4.69	2	Oryza sativa	TVDVEELTVEER
	Glycosyltransferase	2.78	2	Arabidopsis	YVDAVMTIPK
	Phosphoserine aminotransferase	2.35	2	Oryza sativa	FGLIYAGAQK
	NFA2	3.31	2	Arabidopsis	ALGTEIEWYPGK

^1^“Names” were re-annotated in Uniport database, and “uncharacterized protein” were deleted. ^2^Only unused ≥ 2 were shown.

**FIGURE 7:**
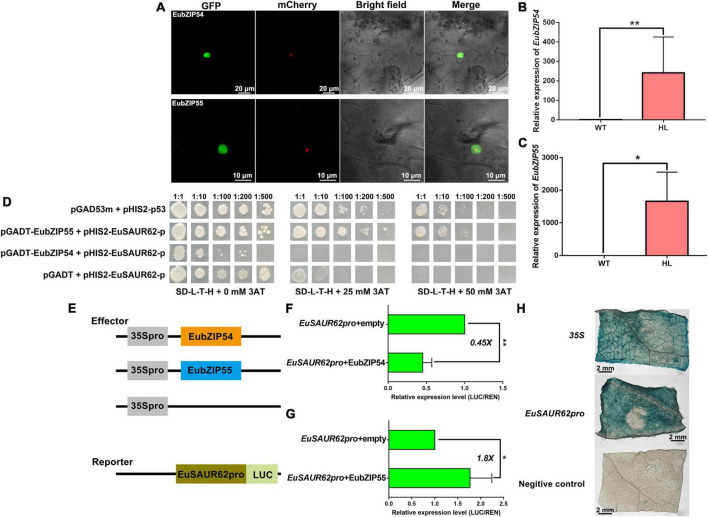
EubZIP55 activates expression of *EuSAUR62*. **(A)** The subcellular localization of EubZIP54/55 in tobacco leaves. EubZIP54/55 were fused to GFP and transfected into tobacco leaves. Merged images including bright field, H2B-mCherry, and GFP fluorescence were observed using a confocal laser scanning microscope. Relative expression of *EubZIP54* **(B)** and *EubZIP55* **(C)** in the fruits of WT and HL was detected using qRT-PCR. **(D)** Yeast one-hybrid assay for the DNA-protein interaction between the *EuSAUR62pro* and EubZIP54 or EubZIP55. The indicated constructs were transformed into yeast reporter strain Y187 and grown on SD–L-T-H media with different concentrations of 3-AT. pGADm53 vs. pHIS-p53 was used as a positive control, and pGADT7 vs. pHIS-*EuSAUR62pro* was used as a negative control. Schematic design is shown on the left **(E)**. Transcription repression and activation activity of EubZIP54 **(F)** and EubZIP55 **(G)** on *EuSAUR62*, respectively, which is expressed by the ratio of firefly luciferase to Renilla luciferase (LUC/REN). **(H)** GUS activity of *EuSAUR62pro* and 35S promoter was compared in tobacco leaves (Scale bar = 2 mm). *p35S:GUS* was used as a positive control, and *empty:GUS* was used as a negative control. Two-tail Student’s *t*-test was used for statistical analysis; Data are mean ± SD. *p < 0.05, **p < 0.01. *EuSAUR62pro*, *EuSAUR62* promoter; HL, hybrid Euryale lines; WT, wild type (North Gordon Euryale).

Both EubZIP54/55 proteins were expressed in the cell nucleus (Figure 7A). The expression of *EubZIP55* and *EubZIP54* in HL was much higher than that in WT. Expression of *EubZIP55*, *EubZIP54*, and *EuSAUR62* were all significantly up-regulated in HL (Figures 4C, 7B, C), indicating that both EubZIP55 and EubZIP54 regulate the expression of *EuSAUR62*. To verify the physical interaction between EubZIP54/55 and *EuSAUR62pro*, we performed a Y1H assay. As shown in Figure 7D, EubZIP55 strongly interacted with the *EuSAUR62pro*, and EubZIP54 formed a weak binding. Then, we performed transient transcription assays using LUC to examine the transcriptional activity of EubZIP54/55 on *EuSAUR62*. The dual reporter vector contained the *EuSAUR62pro* fused with the firefly luciferase reporter gene, with Renilla luciferase driven by CaMV35S as an internal control; the effector vector contained the EubZIP54/55 coding sequence driven by CaMV35S (Figure 7E). As shown in Figure 7G, EubZIP55 activated the expression of *EuSAUR62* 1.8 times, which was indicated by an increase in the firefly luciferase to Renilla luciferase (LUC/REN) ratio compared with that expressing the empty effector vector. In contrast, EubZIP54 repressed the expression of *EuSAUR62* 0.45 times (Figure 7F). In addition, GUS activities were the similar for *EuSAUR62pro* or CaMV35S promoter (Figure 7H), indicating that the promoter of *EuSAUR62* was more easily activated *in vivo*. Results further suggested that *EuSAUR62* is activated mainly by EubZIP55, while EubZIP54 may function as a repressor in cooperation with EubZIP55 to keep the expression of *EuSAUR62* at a normal level.

**FIGURE 8:**
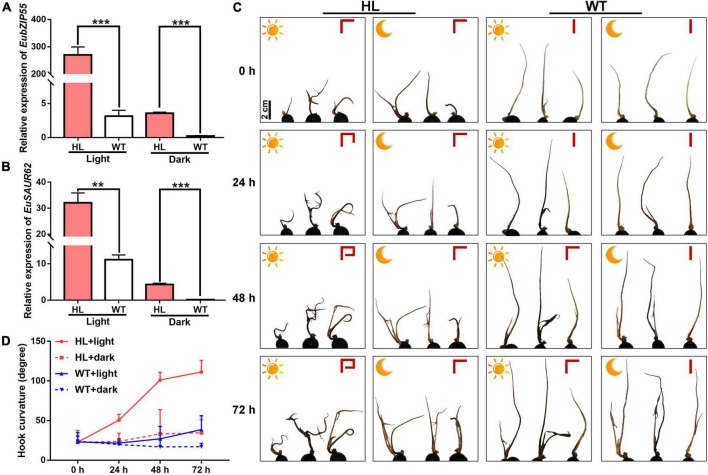
Functional characterization of *EubZIP55* on the auxin-mediated apical hook of Euryale seedlings. Relative expression of *EubZIP55* **(A)** and *EuSAUR62* **(B)** in seedlings of WT and HL under constant light or dark conditions. **(C)** Phenotypes of apical curvature in the 2-week-old seedlings of WT and HL under constant light or dark conditions. The sun represents constant light treatment, and the moon represents dark treatment. Curvature degree is shown as a perpendicular line in the upper right of each picture. Scale bar = 2 cm. **(D)** The quantitative assessment of apical curvature of seedlings. Two-tail Student’s *t*-test was used for statistical analysis; Data are mean ± SD. **p < 0.01, ***p < 0.001.

Previous studies have shown that GBF3 shows light-regulated expression, whereas GBF2 translocates into the nucleus upon light treatment (Yamamoto and Deng, 1999). As we all know, plant phototropism induces the hook curvature of seedlings *via* auxin asymmetrical distribution (Ding et al., 2011). To investigate whether EubZIP55 regulates the light-induced asymmetrical distribution of IAA, we treated HL and WT seedlings under constant light or dark conditions to observe the hook curvature phenotype. The degree of curvature was represented as a perpendicular line in the upper right of Figures 8C,D, during 3 days light treatment, apical hooks of HL were observed to be more curved than that of WT. Although dark treatment inhibited the hook curvature in both types, HL still generated hooks with a slightly more pronounced curve than WT in the dark, indicating that the auxin location was remarkably changed in HL under light conditions. We speculated that the stronger hook curvature in HL was attributed to the high expression of *EuSAUR62* and *EubZIP55* under light conditions. Thus, the expression levels of *EuSAUR62* and *EubZIP55* in these seedlings were determined by qRT-PCR. As expected, it was found that their expression in HL is significantly elevated following light treatment for 3 days compared to that in the dark, and both *EuSAUR62* and *EubZIP55* are more highly expressed in HL than in WT under constant light or dark conditions (Figures 8A,B). Overall, our results suggested that EubZIP55 up-regulates the expression of *EuSAUR62* and alters the distribution of IAA under light stimulation.

## Discussion

### Hybrid Euryale lines promote the enlargement of cells by distributing indole-3-acetic acid around the ovary, which contributes to larger seeds

Euryale is an economic crop, providing food and medicine resources for many people in China and India (Jha and Sharma, 2010; Jha et al., 2018; Zhang et al., 2019). Biomass is a crucial agronomic trait in Euryale, but the molecular mechanisms that regulate it remain unclear. To improve the biomass and yield of Euryale, we generated HL after selecting dominant lines and crossbreeding for many years (Huang et al., 2020). Compared to the WT, HL produced larger and heavier seeds (Figure 1) with a 35% elevation in yield. Seeds of HL also retained the potential medical effects, as some metabolites related to the pharmacologic effects of *Euryale ferox* remained unchanged, such as catechin and hyperoside (Table 1; Wu et al., 2013). Comparing the metabolites between two lines, distinct in the tryptophan metabolism pathway and concentration of IAA forced us to focus on the IAA homeostasis in Euryale (Figure 2). Our previous study showed that the concentration of IAA in fruits is similar at the same growth stages in two lines, while the location of IAA is different. In particular, IAA is mainly distributed in the exocarp in WT and is enriched around the ovary in HL during reproductive growth (Huang et al., 2020). In this study, our histological analysis revealed that cells around the ovary are longer in HL, which supports our opinion that HL distributes its IAA around the ovary to promote cell elongation and ovary enlargement during reproductive growth, thereby resulting in larger seeds (Figure 3).

### *EuSAUR62* regulates indole-3-acetic acid distribution by interacting with PINs

As we concluded above, IAA homeostasis is regulated differently in WT and HL during fruit growth. The IAA homeostasis depends on a complex interplay between IAA metabolism and IAA transport (Rosquete et al., 2012). To understand the molecular mechanisms underlying this regulation, *SAUR* family genes were analyzed because some of them have been reported to mediate both auxin synthesis and its transport in rice (Xu et al., 2017). Among *SAURs* in Euryale, *EuSAUR62* was selected due to its differential expression in HL and WT. *SAURs* are the largest family of early auxin response genes, but there have been few functional analyses to date (Ren and Gray, 2015). Previous studies have implicated that AtSAUR62 promotes cell expansion and proliferation in Arabidopsis (He et al., 2018). Therefore, we speculated that *EuSAUR62* may promote cell elongation due to its high homology with AtSAUR62 (Figure 4A). To confirm whether *EuSAUR62* regulates seed size by mediating the distribution of IAA in Euryale, we generated *EuSAUR62* gain-of-function rice. *OE-EuSAUR62* lines produced longer and heavier grains than WT, but had no effect on the grain width (Figures 5A,D,E and Supplementary Figure 4B), indicating a role for *EuSAUR62* in promoting cell elongation. To further confirm its functions, glumes and outer glume cells were observed. Compared to the WT, glumes in HL were longer and their outer glume cells were significantly elongated (Figures 5B,C,F,G). In addition, *EuSAUR62* also increased plant height and leaf angle by promoting the elongation of parenchyma and pulvinus cells (Supplementary Figure 5). Based on these results, we confirmed that *EuSAUR62* promotes cell elongation during plant growth.

Among all plant hormones, auxin has a unique property, as it undergoes directional, cell-to-cell transport facilitated by auxin carrier proteins (Harrison, 2017). Among these proteins, the PIN family plays a prominent role in regulating auxin efflux due to the asymmetric subcellular localization of their proteins (Hernandez-Hernandez et al., 2018). As we concluded previously, HL distributes its IAA around the ovary to promote seed development, we subsequently asked if *EuSAUR62* mediates localization of IAA. Then, the immunofluorescence assay confirmed that IAA was much more enriched in the ovary in *OE-EuSAUR62* lines compared to the WT (Figure 5H). Further, prediction from the STRING database suggested that EuSAUR62 regulates IAA location in rice by interacting with OsPIN9 proteins, as it has not been reported that *SAURs* directly regulate IAA distribution. The BiFC, mY2H, and Co-IP assays confirmed their interaction (Figure 6). Although the function of OsPIN9 has not been reported yet, the expression pattern in tissues and phylogenetic analysis indicated that it plays a distinct role in regulating auxin-driven organ development (Balzan et al., 2014; Bennett et al., 2014). We speculated that EuSAUR62 might affect the phosphorylation level of OsPIN9, which affects the IAA distribution in rice. However, post-translational regulation between these two proteins needs further investigation. In this study, we did not apply NPA to verify our hypothesis, that is because, although NPA leads to inhibition of auxin export activity by interacting directly in a high-affinity manner with PINs, it also influences the expression level or stability of *EuSAUR62* mRNA (Figures 4F,G; Abas et al., 2021). Genes involved in IAA biosynthesis are also important for its distribution, such as *amidase 1 (AMI1)*, *tryptophan aminotransferase of Arabidopsis 1 (TAA1)*, *nitrilase (NIT)*, *YUCCAs (YUC)*, but did not express differently between the WT and HL at the same growth stage (Supplementary Figure 6). The fact that *OsPIN9* is not induced by IAA suggests that some signaling molecules may be involved in the IAA response and transport of OsPIN9 (Wang et al., 2009). Rapid response of *EuSAUR62* in IAA concentration (Figures 4D,E), and interaction between EuSAUR62 and OsPIN9 proteins (Figure 6) make us conclude that EuSAUR62 functions as a signaling molecule linking IAA and OsPIN9 at the protein level. Specifically, a slight alteration in IAA level induces changes in expression of *EuSAUR62*, which affects IAA distribution by interacting with PIN-FORMED proteins. However, the function of OsPIN9 should be investigated further.

### EubZIPs regulate the expression levels of *EuSAUR62* in light conditions

To understand the underlying regulation in *EuSAUR62* expression, we first confirmed that there is no mutant in *EuSAUR62* sequences between HL and WT (Supplementary Figure 8). Then, we speculated that the different expression of *EuSAUR62* was induced by up-stream TFs. Thus, we performed a DNA-pulldown assay to investigate the TFs that directly bind to *EuSAUR62pro*. A large number of histone modification-relative proteins were detected in LC-MS/MS assay (Table 2), indicating that epigenetic modification mediates the expression of *EuSAUR62*. Normally, histone modification is recruited by TFs to enhance or repress its function in regulating gene expression (Field and Adelman, 2020), further suggesting the existence of at least one TF in regulating the expression of *EuSAUR62*. Combined with JASPAR and transcriptome data, EubZIP55 and EubZIP54, detected in the DNA-pulldown assay, were chosen for further analysis. Expression of both *EubZIP55* and *EubZIP54* were much higher in HL compared to the WT (Figures 7B,C). The Y1H assay indicated a strong interaction between EubZIP55 and the *EuSAUR62pro* (Figure 7D). Interestingly, EubZIP55 activated the expression of *EuSAUR62*, but EubZIP54 repressed it (Figures 7F,G). In the GUS activity assay, *EuSAUR62pro* was activated *in vivo* (Figure 7H), suggesting that the expression of *EuSAUR62* is mainly up-regulated by EubZIP55, and EubZIP54 might act as a repressor to keep the expression of *EuSAUR62* at a normal level.

A previous study placed the AtbZIP proteins into ten groups: A to I, and S (Jakoby et al., 2002). Both AtbZIP55 and AtbZIP54 belong to the G group, denoting that light activation has been proven to stimulate their subcellular localization into the nucleus (Terzaghi et al., 1997). It has been reported that apical hook development is a suitable model for further promoting the mediation of light and the location of IAA on plant growth regulation, as auxin-response and auxin-transport proteins jointly define the spatial and temporal accumulation of auxin during apical hook development (Vandenbussche et al., 2010; Zadnikova et al., 2010, 2016; Beziat et al., 2017; Beziat and Kleine-Vehn, 2018). Euryale is an aquatic plant whose seedlings exhibit auxin-mediated apical curvature under light conditions. We speculated that the EubZIP55-EuSAUR62 module might enhance apical curvature in HL under light treatment. Thus, we treated HL and WT seedlings under constant light or dark conditions to observe the degree of the hook curve. As expected, light significantly enhanced the degree of hook curve (Figures 8C,D) and up-regulated the expression of *EubZIP55* and *EuSAUR62* in HL, while this up-regulation in WT was much lower (Figures 8A,B). In addition, *EubZIP55* and *EuSAUR62* were more highly expressed in HL under light or dark conditions than in the WT (Figures 8A,B). Results revealed that the light-mediated apical hook of Euryale is regulated by the polar distribution of IAA, which is controlled by *EubZIP55* and *EuSAUR62*. Therefore, in this study, we found that it is *EubZIP55* that mediates light-induced expression of *EuSAUR62* in Euryale. We did not focus on the repressor factor EubZIP54 because the underlying mechanism involved in the competition of EubZIP55 and EubZIP54 needs to be further clarified. Extensive studies have reported that the ELONGATED HYPOCOTYL5 (HY5), a bZIP-type transcription factor, tightly controls the light response and photomorphogenesis (Nawkar et al., 2017; Li H. M. et al., 2020; Ortigosa et al., 2020). GBF3 mRNA is accumulated by light exposure in a similar manner to HY5, suggesting that it also participates in the early light response (Kurihara et al., 2020). However, the underlying mechanism of light response of *EubZIP55* in Euryale needs further investigation.

**FIGURE 9:**
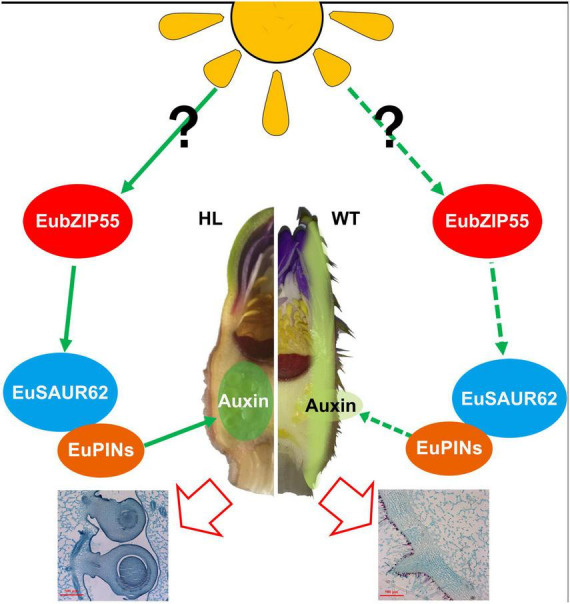
A working model of the role of EuSAUR62 in regulating Euryale seed size. In HL, light increases the expression of EubZIP55, contributing to the higher expression level of *EuSAUR62*, which regulates the localization of auxin by interacting with PINs, thereby promoting ovary enlargement and increasing seed size. In the WT, free-localization of auxin on the exocarp weakens the regulation of seed development and probably promotes the development of pricks.

Overall, we highlighted a molecular bridge role for *EuSAUR62* between light and IAA distribution in Euryale. Specifically, the light activates the expression of *EubZIP55* and subsequently up-regulates the expression of *EuSAUR62*, which affects the distribution of IAA by interacting with PIN family proteins, thereby promoting ovary enlargement and increasing seed size. However, this molecular mechanism is not activated in WT, which potentially results in the development of pricks on the exocarp. The presumed model is shown in Figure 9.

## Data availability statement

The original contributions presented in the study are included in the article/Supplementary material, further inquiries can be directed to the corresponding author.

## Author contributions

Q-NW designed the study. Z-HH, SZ, and W-WT interpreted the study results. Z-HH, KB, Z-HJ, H-FD, and QW performed the research. Z-HH wrote the manuscript. All authors approved the final manuscript.

## Footnotes

1
https://www.arabidopsis.org/

2
http://www.ricedata.cn/gene/index.htm
