# Supplementary material for: Euryale Small Auxin Up RNA62 promotes cell elongation and seed size by altering the distribution of indole-3-acetic acid under the light
Source: Front Plant Sci. 2022 Sep 9;13:931063. doi: 10.3389/fpls.2022.931063 (PMC9500450; doi:10.3389/fpls.2022.931063)
Supplement: Supplementary file 1 [file Data_Sheet_1.docx]

Supplementary Material

## Supplementary tables

**Supplementary table 1.** putative sites were predicted in *EuSAUR62* promoter by JSAPAR.

| Model name | Score | Relative score | Start | End | Strand | predicted site sequence |
| --- | --- | --- | --- | --- | --- | --- |
| MNB1A | 8.115 | 1.00 | 282 | 286 | 1 | AAAGC |
| PBF | 8.062 | 0.99 | 282 | 286 | 1 | AAAGC |
| Dof2 | 8.760 | 0.99 | 483 | 488 | -1 | AAAGCA |
| MNB1A | 8.115 | 1.00 | 484 | 488 | -1 | AAAGC |
| PBF | 8.062 | 0.99 | 484 | 488 | -1 | AAAGC |
| BZIP60 | 13.943 | 1.00 | 917 | 924 | -1 | TGACGTCA |
| BZIP60 | 13.943 | 1.00 | 917 | 924 | 1 | TGACGTCA |
| TGA6 | 15.564 | 1.00 | 917 | 926 | -1 | GATGACGTCA |
| Dof2 | 8.760 | 0.99 | 1509 | 1514 | -1 | AAAGCA |
| MNB1A | 8.115 | 1.00 | 1510 | 1514 | -1 | AAAGC |
| PBF | 8.062 | 0.99 | 1510 | 1514 | -1 | AAAGC |

## Supplementary table 2. Primers used in this study

| **Primer name** | **Sequence** |
| --- | --- |
| *EuSAUR62-F* | GCTCCCACTTGCTTTGCTTG |
| *EuSAUR62-R* | TTAAACCACACTGTGGAGCG |
| *SAUR-r2* | GAATCACAAGGAAAAGTAATCGC |
| *SAUR-r3* | GCCATCTCCACTACTCTCGTTGT |
| *SAUR-r4* | GCGTGTGTCTGTGTTGATGGATA |
| *1301-EuSAUR62-F* | TCCTCTAGAGTCGACCTGCAGTGGCTAGATCATGGTAAGTCA |
| *1301-EuSAUR62-R* | TTACCCTCAGATCTACCATGGTCTTGCAAGATGAGGAAGAC |
| *1300-EuSAUR62-F* | GGGGTACCATGATCAGCACAACGAGAGT |
| *1300-EuSAUR62-R* | GCTCTAGAAACCACACTGTGGAGCGAGT |
| *1300-EubZIP54-F* | CGACTCTAGAAAGCTTATGGGAAACACAGCAGGAGG |
| *1300-EubZIP54-R* | TGCTCACCATGGTACCGGCCACAGCATCAGTACGC |
| *1300-EubZIP55-F* | CGACTCTAGAAAGCTTATGGGCACGGGAAACACAG |
| *1300-EubZIP55-R* | TGCTCACCATGGTACCTACCTGCTGCCAGAGTTCAGAT |
| *1300-OsPIN9-F* | CGACTCTAGAAAGCTTATGATTACGGGTTCGGAGGT |
| *1300-OsPIN9-R* | TGCTCACCATGGTACCCAGCCCCAACAGAATATAGTAAAC |
| *pGREEN800-EuSAUR62pro-F* | CGGTATCGATAAGCTTAGTGGCTAGATCATGGTAAGTCA |
| *pGREEN800-EuSAUR62pro-R* | TAGAACTAGTGGATCCTCTTGCAAGATGAGGAAGAC |
| *qRT-PCR-EuSAUR62-F* | TTCAGCAATGCCACCACATG |
| *qRT-PCR-EuSAUR62-F* | ATTGCCCATCTTCCAACAGC |
| *qRT-PCR-EubZIP55-F* | GGATGAGGAGGATGACCAGA |
| *qRT-PCR-EubZIP55-R* | GCCAACAAAGTCCGAGAAAG |
| *qRT-PCR-EubZIP54-F* | GCCTGCCTCCTCTTCTCTCT |
| *qRT-PCR-EubZIP54-R* | CCCTGCTTCTCTCCCTCTCT |
| *qRT-PCR-EuACTIN-F* | GGAGCGGTAAGACACAAGCT |
| *qRT-PCR-EuACTIN-R* | GGACGATCATCTACCGGCAG |
| *BiFC-EuSAUR62-F* | GAGCTTTCGCGAGCTCATGATCAGCACAACGAGAGT |
| *BiFC-EuSAUR62-R* | GATGGATCTTCTAGAAACCACACTGTGGAGCGAGT |
| *BiFC-OsPIN9-F* | GAGCTTTCGCGAGCTCATGATTACGGGTTCGGAGGT |
| *BiFC-OsPIN9-R* | TCCCTCGAGTCTAGACAGCCCCAACAGAATATAGTAAAC |
| *pBT3-EuSAUR62-F* | CAGGAATTCGATATCAATGATCAGCACAACGAGAGT |
| *pBT3-EuSAUR62-R* | CCCCGACATGGTCGAAACCACACTGTGGAGCGAGT |
| *pPR3-OsPIN9-F* | CGGCCTCTCGAGAATATGATTACGGGTTCGGAGGT |
| *pPR3-OsPIN9-R* | GACGGTATCGATAAGCAGCCCCAACAGAATATAGTAAAC |

## Supplementary Figures


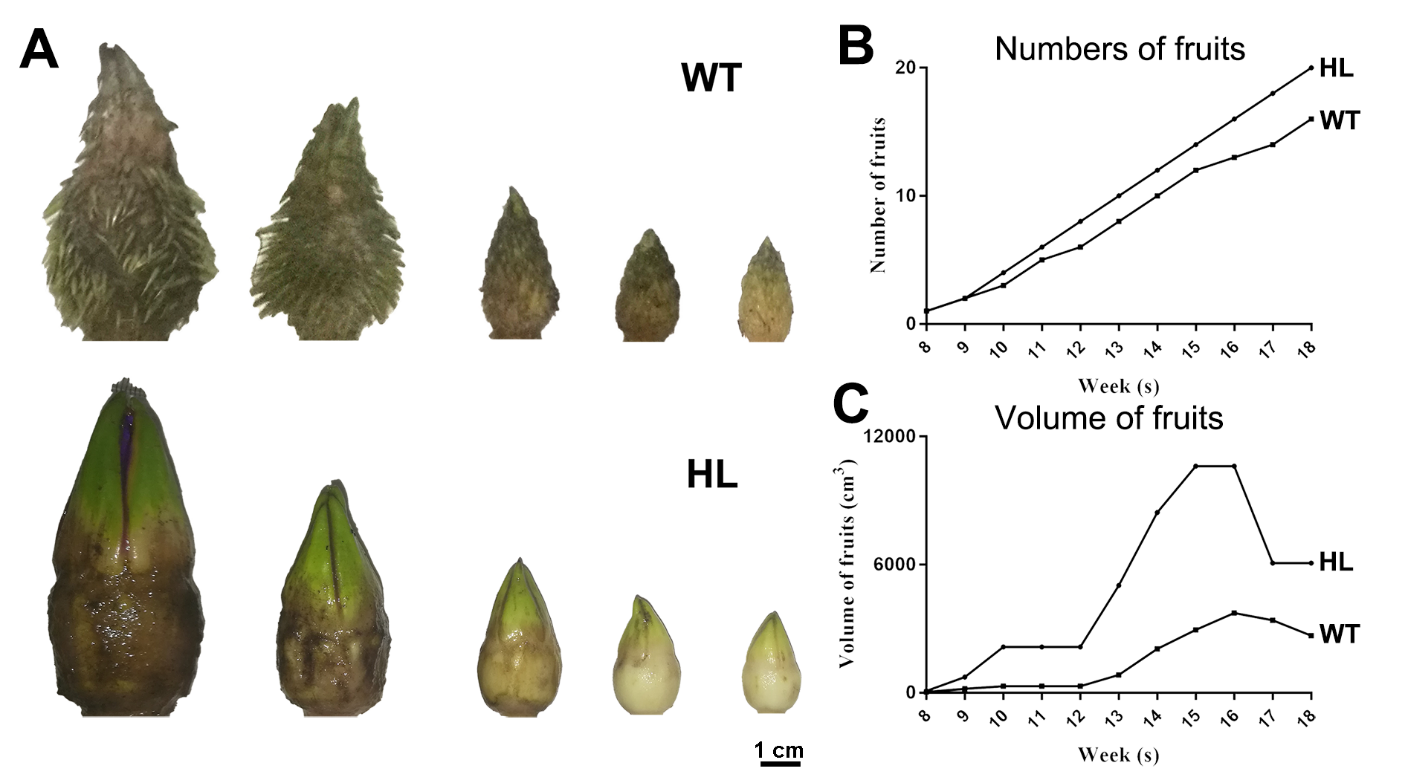


**Supplementary Figure 1.** (**A**) Fruits at different growth stages (Scale bar = 1 cm). left to right: 3-week-old emersed fruit; 3-week-old underwater fruit; 2-week-old underwater fruit; 1-week-old underwater fruit; fruit was less than a week old, all samples were collected in the week 12. Fruit numbers per plant (**B**) and fruit volume (**C**) of two types during reproductive development. HL, hybrid Euryale lines; WT, wild type (North Gordon Euryale).


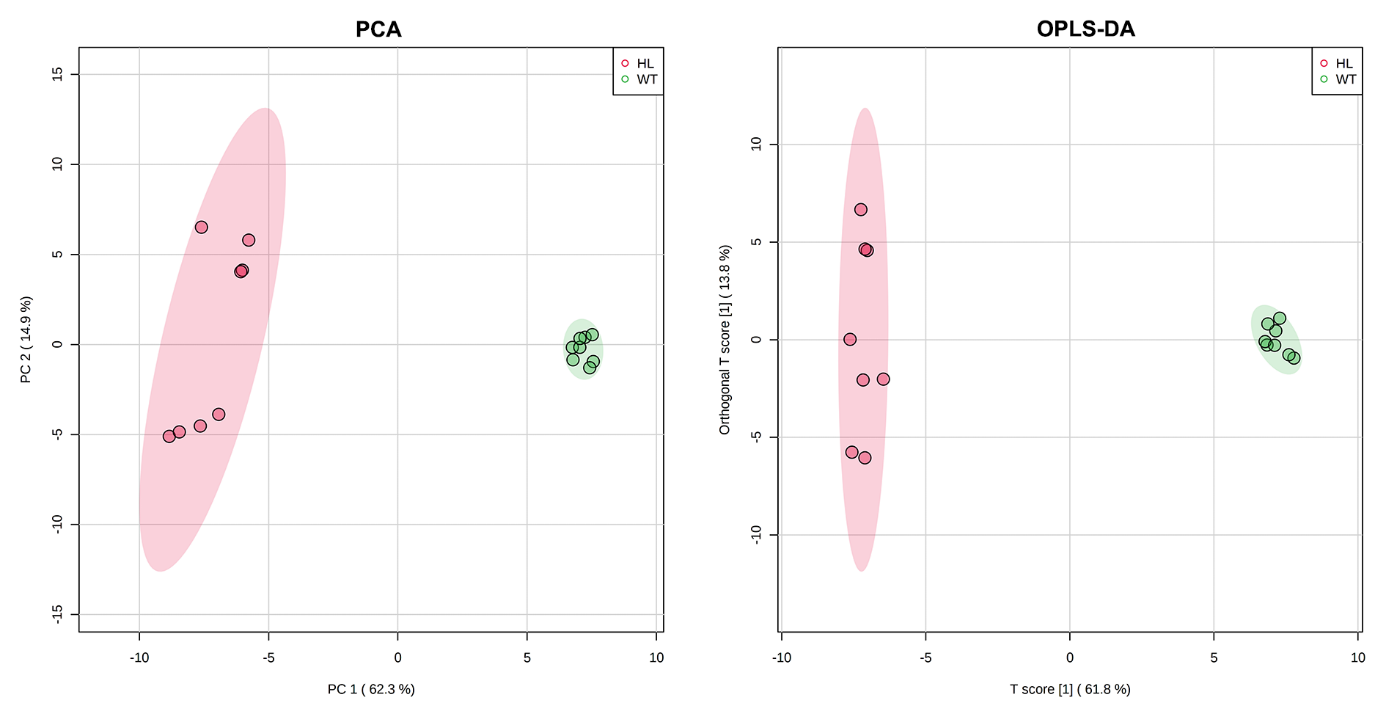


**Supplementary Figure 2.** PCA and OPLS-DA assay on metabolites of the WT and HL.


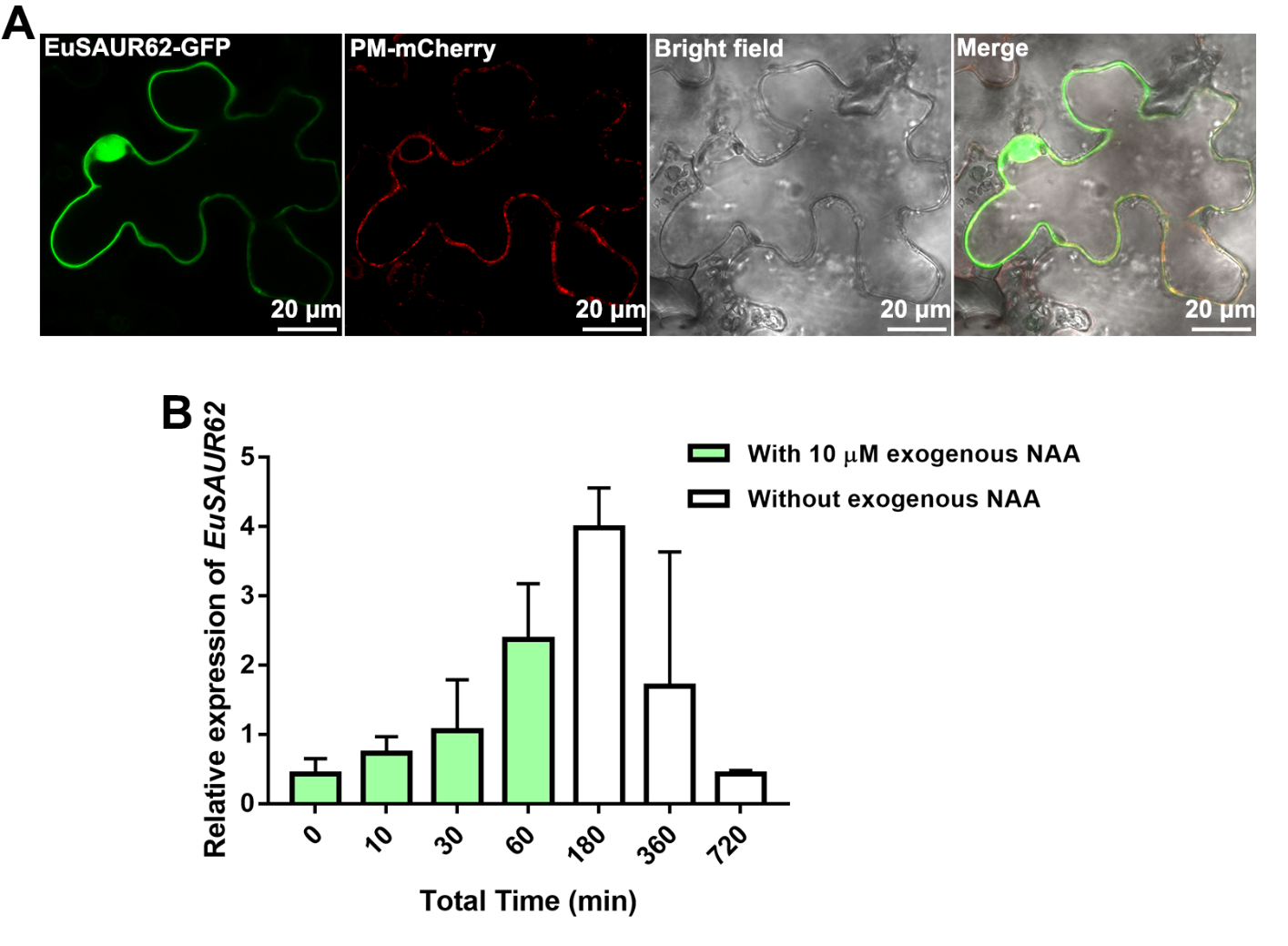


**Supplementary Figure 3.** (**A**) The subcellular localization of EuSAUR62 in tobacco leaves. (**B**) Relative expression of *EuSAUR62* after treating with 10 μM exogenous NAA.


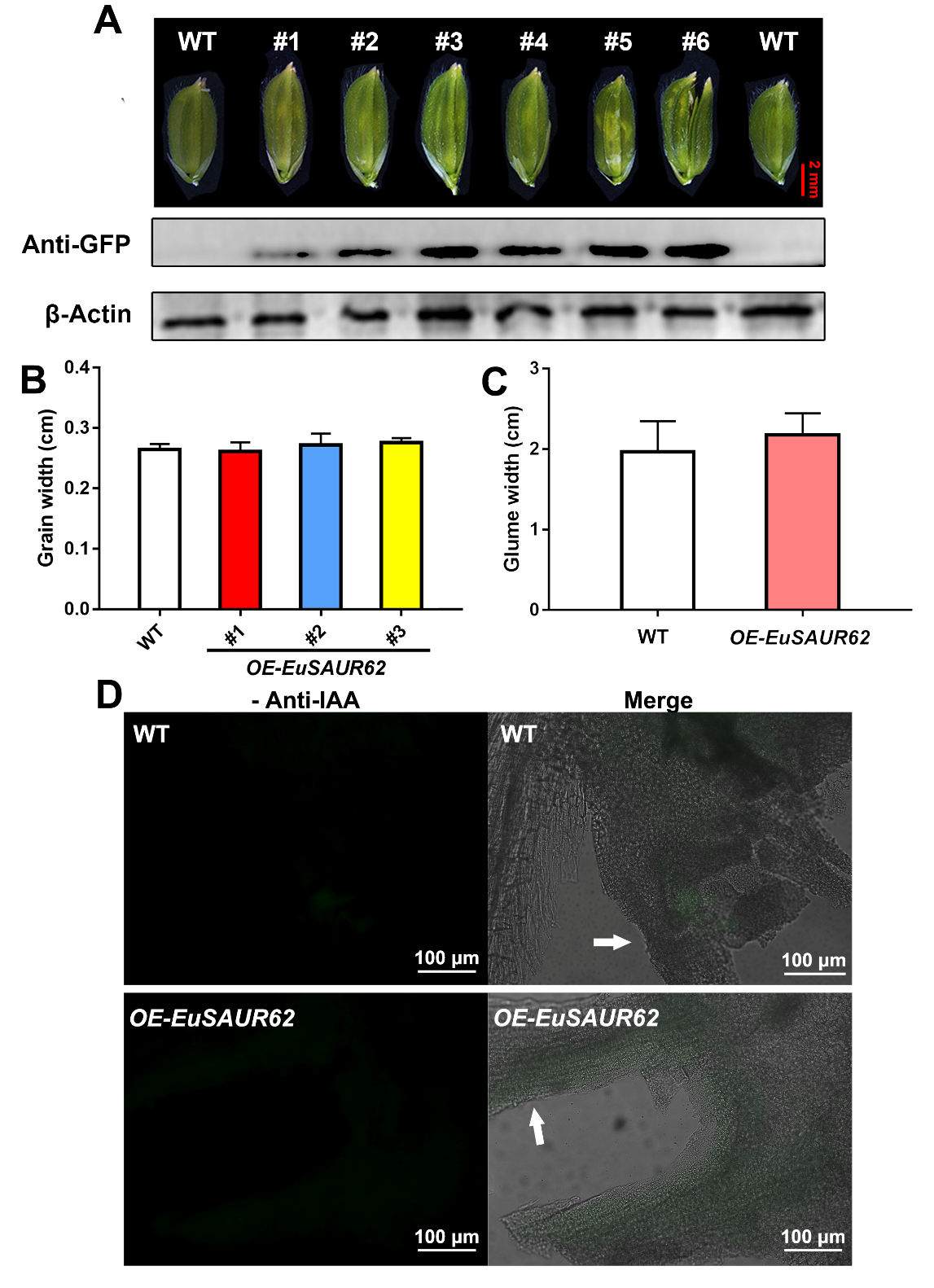


**Supplementary Figure 4.** (**A**) EuSAUR62 protein was detected in fresh grains at heading stage. No significant difference in the grain width (**B**) and glume width (**C**) between the WT and *OE-EuSAUR62*. (**D**) At flowering stage, reproductive tissues incubated with 1% BSA instead of primary antibody were employed as negative control. Rice ovary was denoted with white arrow.


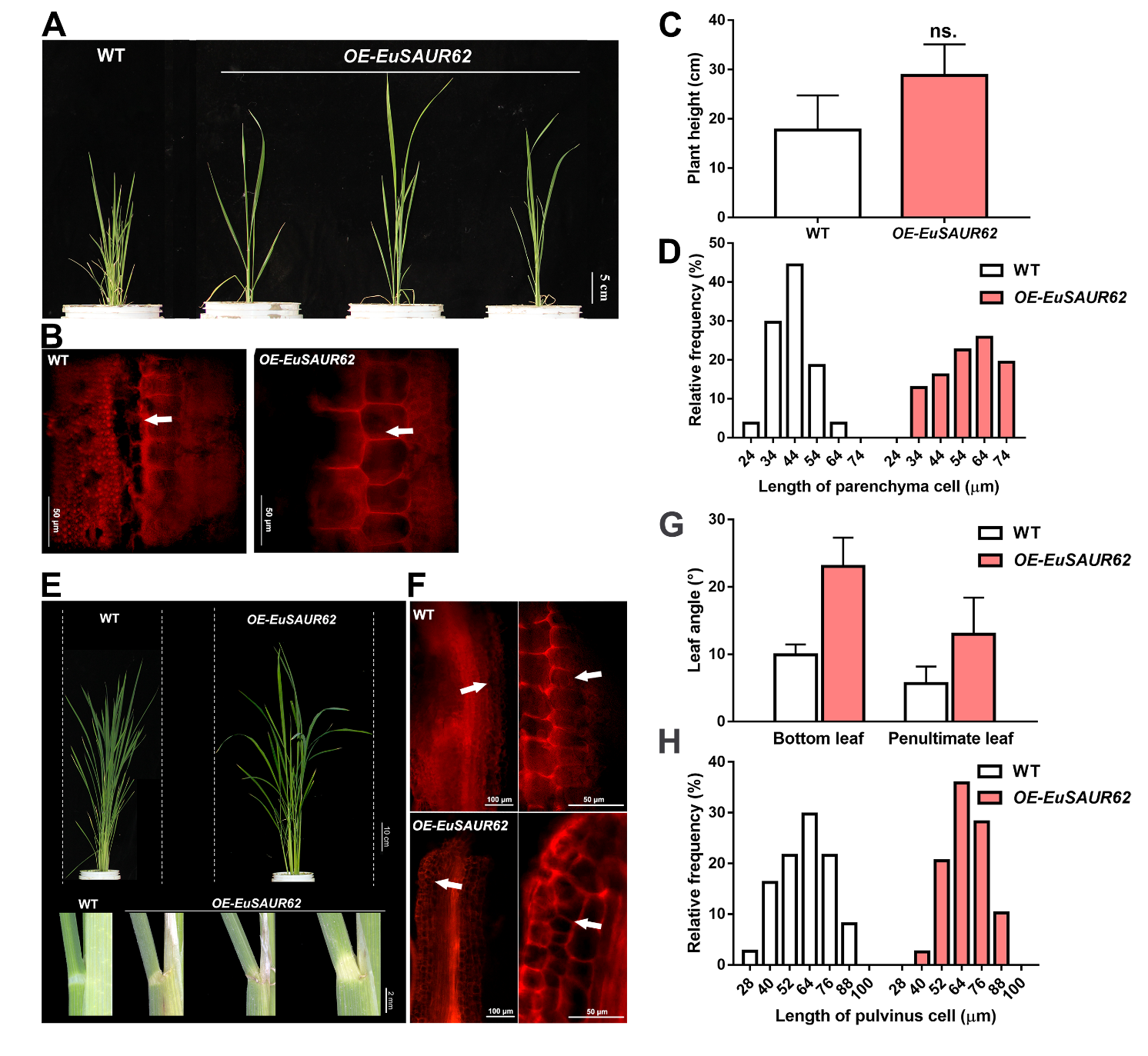


**Supplementary Figure 5. Plant height and leaf angle comparison of the WT and *OE-EuSAUR62* rice.** (**A**) *OE-EuSAUR62* lines increased the plant height 10 days after transplanting compared to the WT. Scale bar = 5 cm. (**B**) Histological observation of red bioluminescence of the parenchyma cells in the young leaves at 10 d. The parenchyma cell was denoted with white arrow. Scale bar = 50 μm. The plant height (**C**) and length frequency distribution of parenchyma cells (**D**) of the WT and *OE-EuSAUR62* plants. (**E**) *OE-EuSAUR62* lines showed loose phenotype, whereas the WT generated compact phenotype (upper). Scale bar = 5 cm. The leaf angle was significantly larger in transformants than in WT (bottom). Scale bar = 2 mm. (**F**) Histological observation of the pulvinus cells of the bottom leaves at 60 d. The pulvinus cell was denoted with white arrow. Scale bar =100 μm (left) and = 50 μm (right), respectively. Leaf angle (**G**) and length frequency distribution of pulvinus cells (**H**) of the WT and *OE-EuSAUR62* plants. Data are mean ± SD. *OE-EuSAUR62*, *EuSAUR62* overexpression lines; WT, wild type rice.


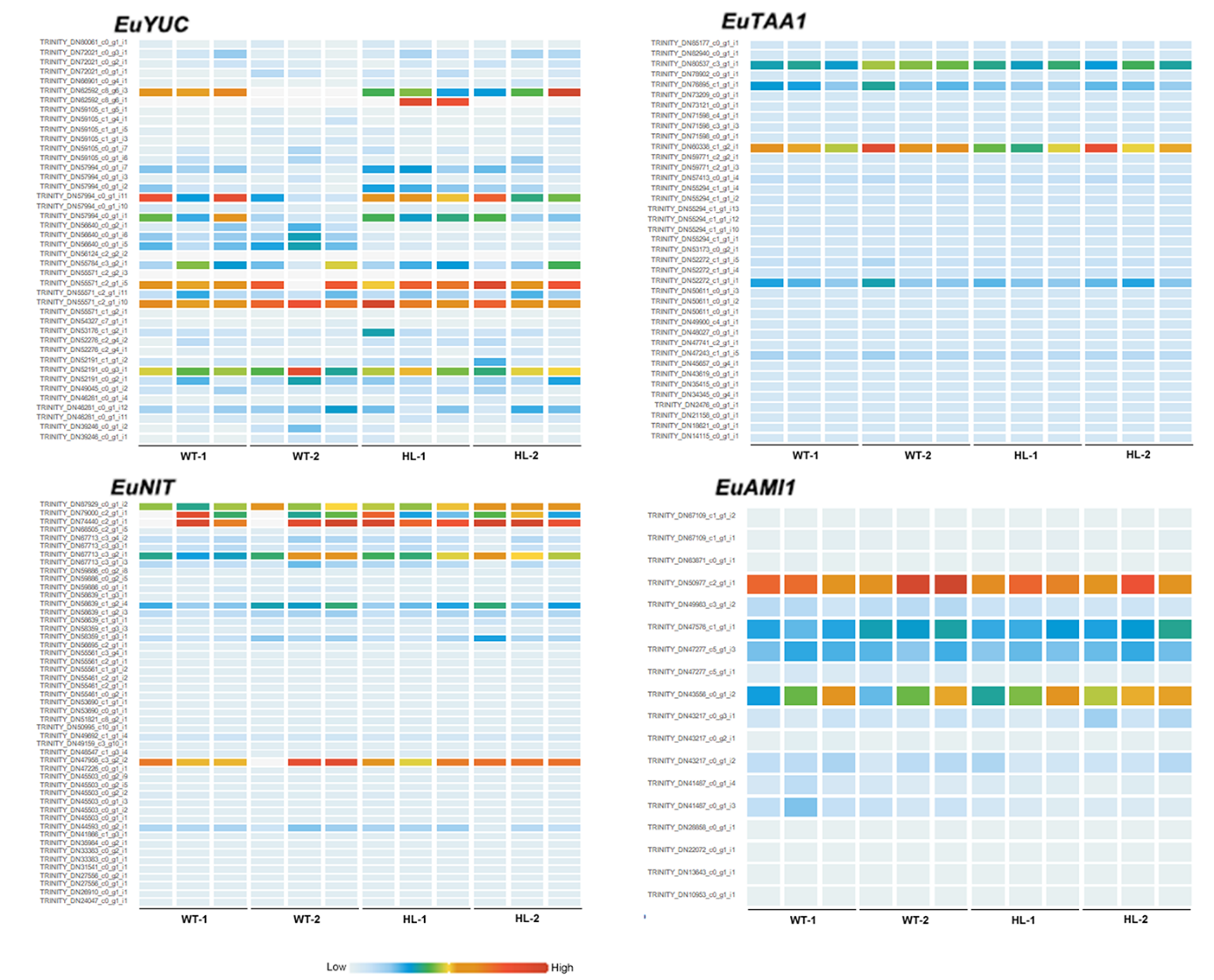


**Supplementary Figure 6.** Expression profiles of all detected Euryale IAA-synthesis genes in transcriptome of young fruits (-1, one-week-old fruits; -2, fruits were less than one week old) collected from 10-week-old WT and HL plants. FPKM value in each sample at different developmental stages was used to generate the heatmap.


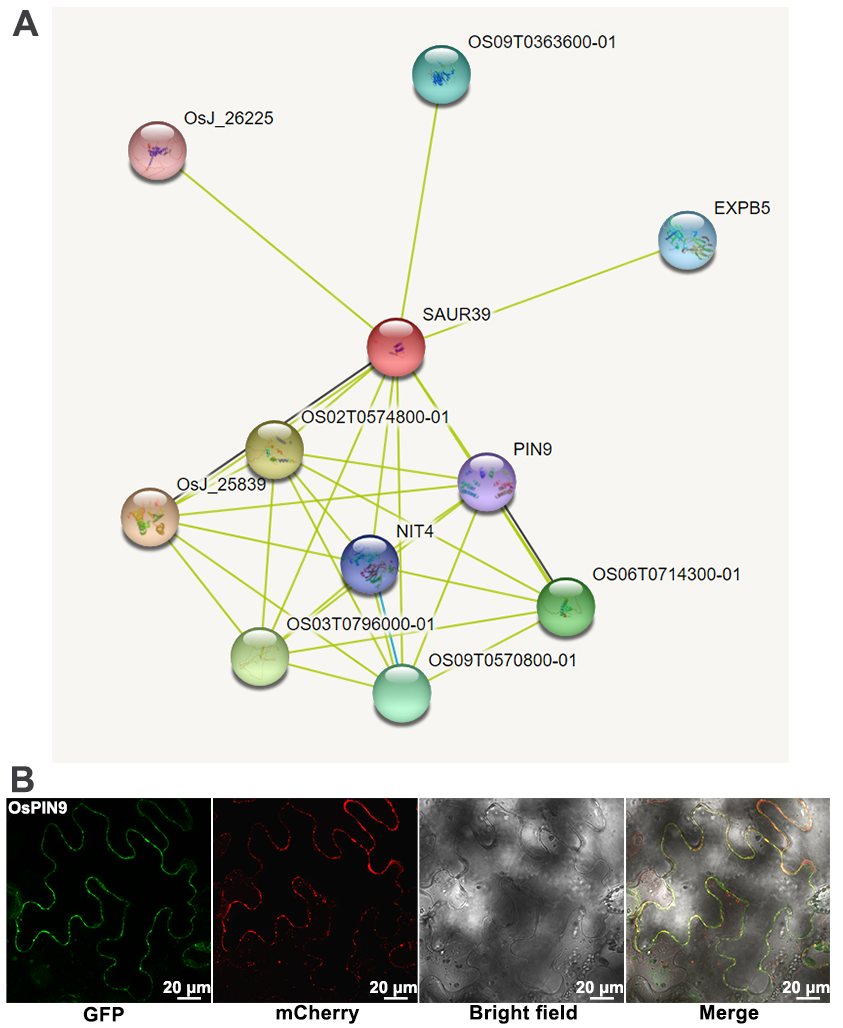


**Supplementary Figure 7.** (**A**) The predicted interaction network of EuSAUR62 in STRING database, OsSAUR39 is considered as the most homologous with EuSAUR62 in this database. (**B**) Subcellular localization of OsPIN9 (Scale bar = 20 μm).


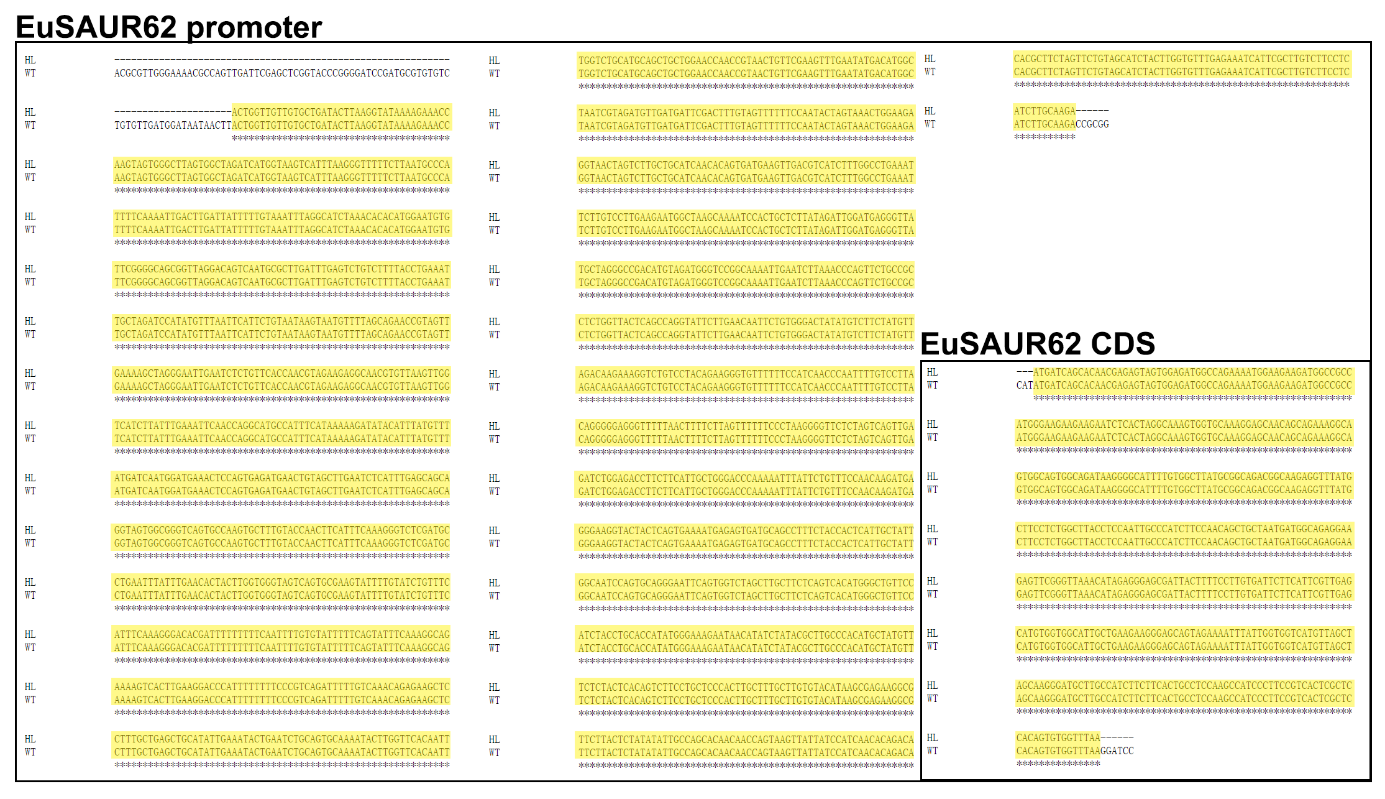


**Supplementary Figure 8.** DNA sequences alignment of *EuSAUR62* promoters and CDS cloned from the WT and HL. The sites highlighted in yellow and marked with asterisk indicate the same DNA sequences.


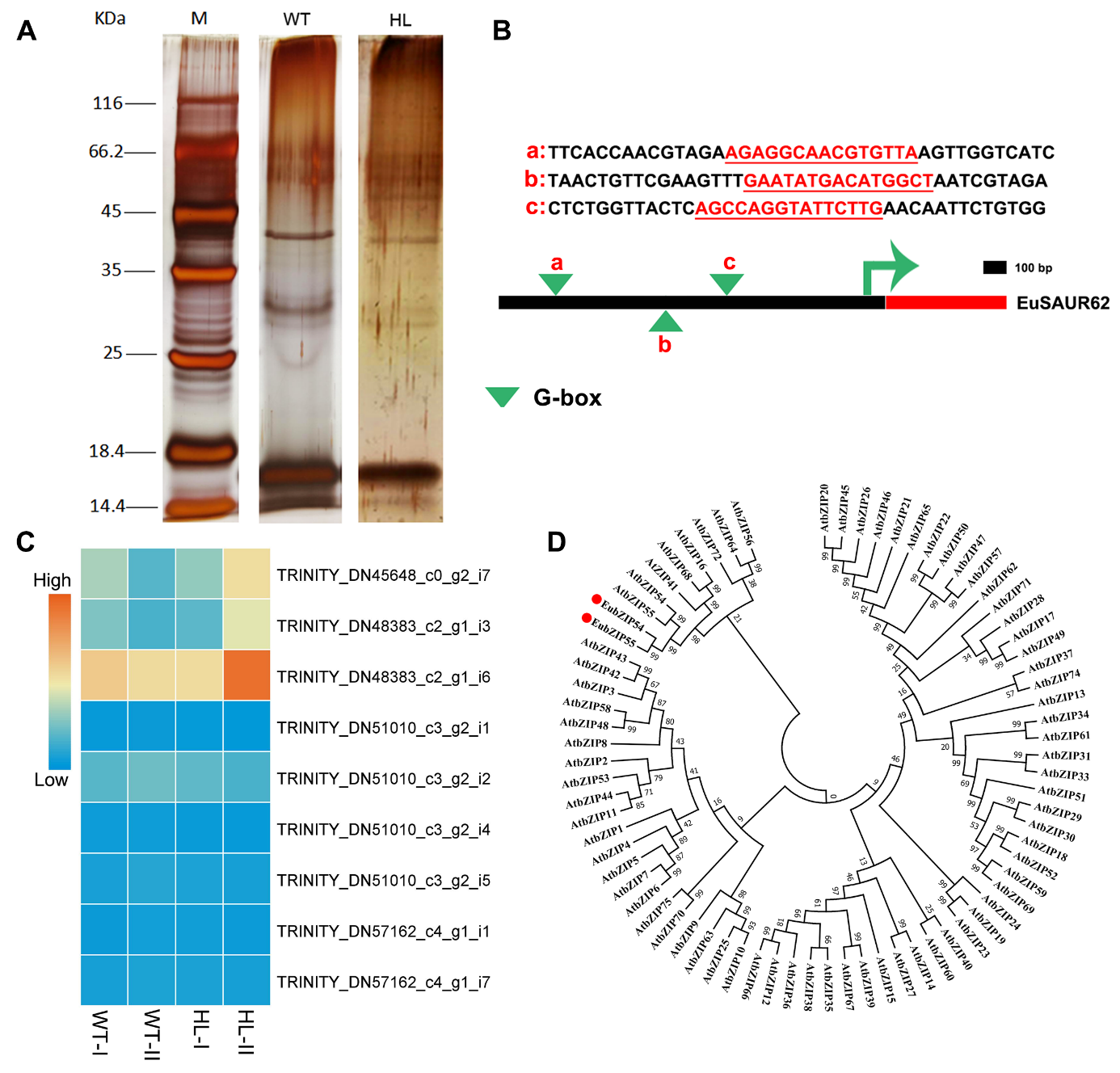


**Supplementary Figure 9.** (**A**) Silver staining of DNA-pulldown products from the WT and HL. (**B**) G-boxes in *EuSAUR62pro*. (**C**) Heat map of all G-box binding factors in Euryale. (**D**) Phylogenetic relationships of EubZIPs and AtbZIPs from *Arabidopsis thaliana*.
